# dsRNA-induced changes in gene expression profiles of primary nasal and bronchial epithelial cells from patients with asthma, rhinitis and controls

**DOI:** 10.1186/1465-9921-15-9

**Published:** 2014-01-29

**Authors:** Ariane H Wagener, Aeilko H Zwinderman, Silvia Luiten, Wytske J Fokkens, Elisabeth H Bel, Peter J Sterk, Cornelis M van Drunen

**Affiliations:** 1 Department of Respiratory Medicine, Academic Medical Center, University of Amsterdam, Meibergdreef 9, 1105 AZ, Amsterdam, The Netherlands; 2 Department of Clinical Epidemiology, Biostatistics & Bioinformatics, Academic Medical Center, University of Amsterdam, Amsterdam, The Netherlands; 3 Department of Otorhinolaryngology, Academic Medical Center, University of Amsterdam, Amsterdam, The Netherlands

**Keywords:** Asthma, Rhinitis, Epithelium, Gene expression, dsRNA

## Abstract

**Background:**

Rhinovirus infections are the most common cause of asthma exacerbations. The complex responses by airway epithelium to rhinovirus can be captured by gene expression profiling. We hypothesized that: a) upper and lower airway epithelium exhibit differential responses to double-stranded RNA (dsRNA), and b) that this is modulated by the presence of asthma and allergic rhinitis.

**Objectives:**

Identification of dsRNA-induced gene expression profiles of primary nasal and bronchial epithelial cells from the same individuals and examining the impact of allergic rhinitis with and without concomitant allergic asthma on expression profiles.

**Methods:**

This study had a cross-sectional design including 18 subjects: 6 patients with allergic asthma with concomitant rhinitis, 6 patients with allergic rhinitis, and 6 healthy controls. Comparing 6 subjects per group, the estimated false discovery rate was approximately 5%. RNA was extracted from isolated and cultured primary epithelial cells from nasal biopsies and bronchial brushings stimulated with dsRNA (poly(I:C)), and analyzed by microarray (Affymetrix U133+ PM Genechip Array). Data were analysed using R and the Bioconductor Limma package. Overrepresentation of gene ontology groups were captured by GeneSpring GX12.

**Results:**

In total, 17 subjects completed the study successfully (6 allergic asthma with rhinitis, 5 allergic rhinitis, 6 healthy controls). dsRNA-stimulated upper and lower airway epithelium from asthma patients demonstrated significantly fewer induced genes, exhibiting reduced down-regulation of mitochondrial genes. The majority of genes related to viral responses appeared to be similarly induced in upper and lower airways in all groups. However, the induction of several interferon-related genes (*IRF3, IFNAR1, IFNB1, IFNGR1, IL28B*) was impaired in patients with asthma.

**Conclusions:**

dsRNA differentially changes transcriptional profiles of primary nasal and bronchial epithelial cells from patients with allergic rhinitis with or without asthma and controls. Our data suggest that respiratory viruses affect mitochondrial genes, and we identified disease-specific genes that provide potential targets for drug development.

## Background

Viral respiratory tract infections are the most common cause of asthma exacerbations [1], with rhinovirus (RV) being the most prominent virus involved. Patients with asthma are not at increased risk of a viral infection as compared to healthy controls, but in case of an upper respiratory infection they are more prone to develop a lower respiratory tract infection with more severe symptoms [2]. This suggests that host characteristics are contributing to the clinical responses to virus infections in asthma.

The majority of patients with asthma is sensitized to environmental allergens, which is associated with allergic airways inflammation [3]. There is increasing evidence of interaction between viral infection and allergic sensitization, with increased risk for a more severe exacerbation if patients with asthma are atopic [4]. Since asthma and rhinitis co-exist to variable degrees [5], the host characteristics in upper and lower airways when encountering a respiratory virus are likely to vary to a similar extent. In order to develop effective interventions for the prophylaxis and treatment of virus-induced exacerbations in asthma, it is mandatory to map the responses of both upper and lower airways to respiratory viruses in patients with asthma and/or rhinitis.

The upper and lower airway epithelial layer is constantly exposed to viruses, bacteria and allergens, and represents the first line of defence. Experiments with nasal and bronchial epithelial cells from the same individuals suggested greater susceptibility of the lower airways to RV because of differences in electrical resistance and viral replication between upper and lower airway epithelium, though no differences were observed between asthma and healthy controls [6]. However, there is increasing evidence that pre-existing asthma affects epithelial cytokine responses to RV, such as impaired interferon (IFN) production and differences in expression of genes involved in immune response and airway remodelling [7-9]. Collectively, these studies suggest that the complex role of the airway epithelium in response to viruses is affected by asthma- and allergy-related changes in gene expression.

Double-stranded RNA (dsRNA) is produced during RV replication and is an important stimulus of the host immune response [10]. Therefore, we hypothesized a) that upper and lower airway epithelium exhibit differential responses to dsRNA infection and b) that this is modulated by the presence of asthma and allergic rhinitis. To that end, we aimed to compare dsRNA-induced gene expression profiles of cultured primary nasal and bronchial epithelial cells obtained from patients with asthma with concomitant allergic rhinitis, patients with allergic rhinitis alone, and healthy controls. The observed gene-expression profiles can be used to delineate the critical pathways that are operative in epithelial cells after virus infection in patients with and without pre-existing airways disease.

## Methods

### Subjects

In total, 18 subjects (>18 y) were recruited for this study, which is part of a larger project [11]. The subjects comprised of three groups: *1)* 6 subjects with allergic asthma with concomitant allergic rhinitis, *2)* 6 subjects with allergic rhinitis, and *3)* 6 healthy controls. Patients with asthma had episodic chest symptoms, controlled or partly controlled disease according to GINA-criteria [12] with airway hyperresponsiveness (PC_20_ methacholine ≤ 8 mg/mL) according to the standardized tidal volume method [13]. All subjects with allergic rhinitis had persistent, moderate to severe disease according to ARIA-criteria [14] with nasal symptoms for more than 4 days a week during more than 4 consecutive weeks. Atopic status was based on the presence of at least one positive skin prick test response (>3 mm wheal) to common allergens. Subjects had refrained from using any medication for their asthma, rhinitis or allergy in the four weeks prior to taking biopsies and brushings. Healthy controls had normal spirometric values without airway hyperresponsiveness (PC_20_ > 8 mg/mL), did not have a history of lung disease and were not atopic.

All subjects were non-smokers or ex-smokers (≥5 pack years). Subjects had not smoked within 12 months prior to the study and they did not have any signs of a respiratory infection at the time of study visits. In the case of a respiratory infection, a 6-week recovery period was taken into account.

The study was approved by the hospital Medical Ethics Committee of the Academic Medical Centre in Amsterdam, and the study was registered in the Netherlands trial register (http://www.trialregister.nl) with identifier NTR2125. All patients gave written informed consent.

### Design

This cross-sectional study consisted of two study visits. At least 14 days after the screening visit for checking inclusion and exclusion criteria, a fiberoptic bronchoscopy was performed during which 4 bronchial brushings were taken. Local anaesthesia of the larynx and lower airways was achieved using 1% lignocaine. Subsequently, 4 nasal biopsies were taken from the lower edge of the inferior turbinate. Local anaesthesia was achieved by application of adrenalin and cocaine under the inferior turbinate without touching the biopsy site.

### Poly(I:C) stimulation of cultured primary epithelial cells

Epithelial cells were isolated from bronchial brushings and nasal biopsies as previously described [11]. The cells were cultured to 80% confluence and pre-incubated with BEBM prior to stimulation. After 24 hours, the pre-incubation medium was removed and cells were exposed to BEBM containing 20 μg/ml Poly(I:C), a synthetic dsRNA, or with BEBM alone (control condition). RNA was extracted from the cells following 24 hours of stimulation. For a detailed description of the primary epithelial cell culture and RNA extraction, see Additional file 1: Methods of the online supplement.

### Analysis and statistics

Microarray analysis was done by previously published method [11]. In short, Human Genome U133+ PM Genechip Array (Affymetrix inc., Santa Clara, CA, USA) was used for microarray analysis of genes. Next, Affymetrix Expression Console was used to analyze the array images using the robust multichip analysis (RMA) algorithm. Normalized data were further analysed using R (version 2.15) and the Bioconductor Limma package [15]. Differential gene expression was measured by empirical Bayes t-statistics and p-values were adjusted for false discovery rate correction [16]. The full microarray data was uploaded to the Gene Expression Omnibus (GEO) with accession number GSE51392.

GeneSpring GX12 (Agilent Technologies, Amstelveen, The Netherlands) was used for Gene ontology (GO) analysis. To investigate the overrepresentation of gene ontology groups we used the p-value adjusted for multiple testing by Benjamini-Yekutieli [17] (p-value <0.01). To enable validation of the microarray experiment changes in gene expression after poly(I:C) stimulation of nine genes were measured by independent real time PCR on the same starting material used for the microarray analysis (Additional file 1: Methods of the online supplement).

Sample size estimation was performed by a validated algorithm for microarray studies as published previously by our group [18], using data from two studies from others and ourselves [19,20]. This analysis showed that the present study had a False Discovery Rate of approximately 5% for detecting at least a 1.5-fold difference in gene expression when comparing three groups of 6 subjects at a significance level of 0.0001,

## Results

The results of the validation by independent real time PCR are presented in Additional file 1: Table S1 and Additional file 2: Figure S1 of the online supplement.

### Poly(I:C)-induced changes in airway epithelial cells

Sufficient RNA for gene expression profiling was obtained from both nasal and bronchial epithelium of 6 healthy controls, 5 patients with allergic rhinitis (1 patient was excluded because of insufficient RNA in the nasal sample) and 6 patients with both allergic rhinitis and allergic asthma. The baseline characteristics of the subjects included in the study are shown in Table 1[11]. There were no differences between the groups in age and spirometry (*p* = 0.4 and *p* = 0.3, respectively). As expected, PC_20_ was significantly lower in the patients with both asthma and rhinitis as compared to those with rhinitis alone and the controls. None of the healthy controls and only 1 out of 5 patients with rhinitis had a drop of 20% in FEV_1_ at the highest concentration of methacholine-bromide 19.6 mg/ml.

**Table 1:** Baseline characteristics

	**Subjects**
	N = 17
Age*	24 (20–30)
Female gender (n)	14
Prebronchodilator FEV_1_% predicted†	109 (11.0)
PC_20_‡**	0.35 (0.3)

*Median (range).

†Mean (Standard Deviation).

‡Geometric Mean (Geometric Standard Deviation).

**Only from patients with both allergic asthma and rhinitis (none of the healthy controls and only 1 out of 5 patients with rhinitis had a drop of 20% in FEV_1_ at the highest concentration of methacholine-bromide 19.6 mg/ml).

Overall, a strong response was observed after stimulation of the airway epithelial cells with poly(I:C). Using a cut-off of p < 0.05 (adjusted for multiple testing), 10163 and 8342 genes were significantly induced in the healthy upper and lower airway epithelium respectively, 9353 and 5190 genes in patients with allergic rhinitis, and 4919 and 5810 genes in patients with both asthma and allergic rhinitis (Additional file 1: Table S2 of the online supplement). The majority of the most highly up-regulated genes were interferon-related genes (*CCL3, CCL4, CCL5, RSAD2, OAS1, OASL, MX1, MX2, IFI6, IFIT1, IFIT3, IFI44, IFI44L, CXCL10, CXCL11, TNFAIP6*), which were induced in both upper and lower airways in all subjects (Tables 2 and 3).

**Table 2:** Most highly up-regulated genes in the upper airways

	**Healthy**		**Allergic rhinitis**		**Asthma**	
**Gene ID**	**FC**	**adj.P.Val**	**FC**	**adj.P.Val**	**FC**	**adj.P.Val**
CCL5	517.58	2.27E-06	717.90	1.11E-06	148.81	2.63E-02
RSAD2	260.58	6.48E-06	638.34	9.05E-07	98.63	2.63E-02
CMPK2	151.01	5.93E-05	469.49	4.62E-08	81.61	2.63E-02
OASL	256.41	1.48E-06	442.40	9.31E-08	93.45	2.63E-02
CCL3	277.19	3.04E-06	334.76	2.77E-06	45.69	2.78E-02
C4orf7	249.61	3.42E-07	319.09	3.92E-06	31.69	3.62E-02
MX2	115.45	3.43E-05	287.26	8.56E-08	72.32	2.63E-02
CCL4	255.13	2.38E-06	243.83	6.17E-06	39.32	2.82E-02
IFI44L	89.69	1.32E-04	240.00	5.17E-07	58.14	2.82E-02
APOBEC3A	224.06	6.80E-08	235.49	9.36E-07	40.77	2.81E-02
CXCL11	104.60	3.78E-06	189.03	2.90E-06	70.87	2.63E-02
CXCL10	74.79	3.67E-05	175.30	1.38E-06	72.35	2.63E-02
DEFB4A	169.21	2.86E-07	165.40	4.47E-06	51.52	3.06E-02
ESM1	109.38	7.55E-07	166.63	2.77E-06	34.37	2.78E-02
IFI44	64.88	1.56E-04	164.77	2.77E-06	42.51	3.06E-02
OAS1	73.40	3.58E-05	160.01	9.36E-07	38.28	2.79E-02
IFIT1	57.77	4.51E-04	150.43	2.08E-05	40.36	2.81E-02
IFIT3	84.45	2.91E-05	149.76	2.16E-06	41.00	2.63E-02
LAMP3	88.39	1.71E-05	137.82	5.31E-06	29.48	2.75E-02
TNFAIP6	137.00	4.06E-07	102.91	3.25E-05	27.28	3.28E-02

FC = fold change; adj.P.Val = *p*-value adjusted for multiple testing.

**Table 3:** Most highly up-regulated genes in the lower airways

	**Healthy**		**Allergic rhinitis**		**Asthma**	
**Gene ID**	**FC**	**adj.P.Val**	**FC**	**adj.P.Val**	**FC**	**adj.P.Val**
CCL5	736.54	6.09E-08	322.77	1.61E-05	231.87	9.60E-03
CMPK2	395.26	2.56E-08	360.62	6.91E-06	164.40	8.69E-03
RSAD2	363.02	1.47E-07	304.55	6.91E-06	167.47	9.76E-03
CXCL11	315.72	1.08E-07	109.20	1.58E-04	156.95	8.45E-03
IFIT1	307.08	9.93E-08	281.31	2.49E-05	161.84	1.05E-02
IFI44L	274.95	2.91E-08	211.91	1.08E-05	113.56	9.51E-03
CXCL10	263.55	2.97E-06	121.34	5.72E-04	88.92	1.09E-02
OASL	241.59	6.78E-08	138.85	5.11E-05	160.96	8.11E-03
IFI44	236.92	4.06E-09	70.93	1.67E-05	104.82	9.45E-03
MX2	201.46	2.56E-08	163.72	1.64E-05	96.81	8.67E-03
MX1	140.44	1.43E-07	175.11	1.67E-05	67.81	9.45E-03
IFI6	165.88	5.52E-10	123.34	1.24E-05	77.06	9.76E-03
DEFB4A	31.48	3.94E-04	137.90	2.06E-04	25.92	1.11E-02
LAMP3	131.90	2.15E-06	86.23	2.50E-04	50.68	1.39E-02
XAF1	110.43	6.45E-08	98.06	1.67E-05	61.68	7.99E-03
OAS1	109.84	1.13E-07	86.84	5.10E-05	47.06	1.05E-02
APOBEC3A	101.03	3.84E-07	86.19	8.06E-04	74.29	7.44E-03
IFIT3	94.85	9.47E-08	88.51	5.73E-05	56.72	8.56E-03
CCL4	83.19	2.22E-04	20.90	2.27E-02	35.57	1.25E-02
CCL3	69.05	7.38E-04	17.00	4.78E-02	35.62	1.27E-02

FC = fold change; adj.P.Val = *p*-value adjusted for multiple testing.

### Functional characterization

**Figure 1:**
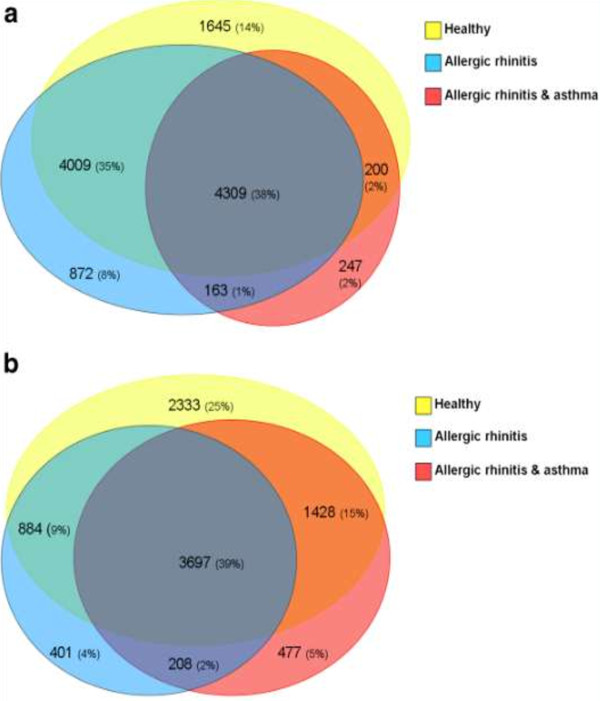
**Venn-diagrams of up- and down-regulated genes. A**. Venn-diagram of genes up- or down-regulated in the upper airways. **B**. Venn diagram of genes up- or down-regulated in the lower airways.

It appeared that ~40% of the poly(I:C)-induced genes was altered in both upper and lower airways in all 3 subject-groups (see Figure 1A and 1B). When studying the functional characterization of these genes, many comparable GO-classes were significantly enriched in the upper and lower airways, *e.g. response to virus*, *apoptotic process*, *antigen processing and presentation of peptide antigen via MHC class I*, and *regulation of I-κB/NF-κB*. The majority of genes related to *response to virus* were induced in all groups in the same direction (up- or down-regulated) (Additional file 1: Table S3A and S3B of the online supplement). Among the genes assigned to this GO-class were those involved in TLR3-signaling (*TLR3, TICAM, TBK1, MYD88, IRAK3*), interferons (*IFNB1, IFNE, IFNK*), various interferon receptors and interferon-induced proteins, cytokines (*IL12A, IL23A, IL6*) and particular cytokines related to type III interferons (*IL28A, IL28B IL29*), chemokines (*CCL22, CCL4, CCL5*), and transcription factors (*IRF3, IRF7, IRF9, RELA, FOSL1*).

#### Upper and lower airways

There were between-group differences in genes related to GO-class *response to virus*. Among the genes that were induced in all subjects except for patients with asthma were interferon involved genes (*IL28B, IFNAR1), CCL22,* and *FOSL1* with respect to the upper airways, and several interferon involved genes (*IRF3, IFNAR1, IFNB1, IFNGR1, IL28B*) in the lower airways.

The upper airways of patients with asthma with concomitant allergic rhinitis demonstrated significantly fewer poly(I:C)-induced genes. In total, 57% of the induced genes was altered exclusively in the upper airways of either healthy subjects or allergic rhinitis patients and not in the upper airways of patients with asthma. Among these genes many GO classes were significantly overrepresented, amongst which many genes were assigned to *metabolic process*, *mitochondrion* and *electron transport chain*. Up to 2000 genes were assigned to *metabolic process* of which 347 genes overlap with class *mitochondrion* (Additional file 1: Table S4 of the online supplement). These mitochondrial genes were mostly down-regulated. The latter class overlaps with genes assigned to the class *electron transport chain*, including several NADH dehydrogenase subcomplexes.

With respect to genes induced in the lower airways, significantly enriched GO-glasses emerged among genes exclusively induced in the lower airways of healthy subjects, including *metabolic process*, *nucleus* and *mitochondrion*. Among these genes, 915 genes were assigned to the GO-class *metabolic process*, of which 616 genes match with class *nucleus* and 191 with class *mitochondrion* (Additional file 1: Table S5 of the online supplement). Along with the upper airways, the GO-class *mitochondrion* was overrepresented among genes induced in the lower airways of both healthy controls and allergic rhinitis patients (Additional file 1: Table S6 of the online supplement). In addition, these mitochondrial genes were primarily down-regulated.

### Disease-specific gene expression induction

We identified genes that were exclusively differentially expressed in patients with asthma and/or allergic rhinitis. We limited to genes that were up- or down-regulated by more than threefold (at least in one of the patient-groups) (see Tables 4 and 5). Among these genes were ciliary gene *BBS1*, nebulette (*NEBL*), nucleoside diphosphate kinase (*NME7*), the ubiquitous protein *AHNAK*, the calcium-binding *S100A7A*, myosin light chain kinase (*MYLK*), the cornulin gene (*CRNN*), filaggrin (*FLG*), complement factor B (*CFB*), bone morphogenetic protein 6 (*BMP6*), adaptor protein SH3KBP1, endoplasmatic reticulum-associated aminopeptidase 1 (*ERAP1*), and lysosome-associated membrane protein 2 (*LAMP2*).

**Table 4:** Disease-specific genes induced in the upper airways

**Gene ID**	**FC rhinitis**	***p*-value rhinitis**	**FC asthma**	***p*-value asthma**	**Disease-specific**
LAMA3	10.87	<0.001	1.62	0.031	Significant *p*-value in patients with allergic rhinitis with or without asthma
SH3KBP1	9.12	<0.001	5.13	0.027	
NME7	6.44	<0.001	2.68	0.031	
ERAP1	5.06	<0.001	3.76	0.026	
MFI2	5	<0.001	3.39	0.042	
UBASH3B	3.45	<0.001	2.4	0.042	
NSD1	3.11	0.001	2.31	0.034	
SLC23A2	−3.55	<0.001	−2.22	0.037	
ARHGAP5	−3.57	<0.001	−2.12	0.039	
ATP6V0A2	−3.79	<0.001	−2.57	0.03	
ANKH	−3.96	<0.001	−2.48	0.03	
WWTR1	−4.03	<0.001	−2.41	0.048	
NEBL	−4.69	0.001	−3.75	0.039	
MYO5A	−4.76	<0.001	−2.67	0.033	
LAMP2	−5.02	<0.001	−3.05	0.035	
AHNAK	−5.02	<0.001	−3.33	0.031	
RAB22A	−5.93	<0.001	−3.18	0.031	
PPAT	−6.37	<0.001	−2.72	0.044	
TFRC	−9.93	<0.001	−5.63	0.028	
CCNB2	−15.44	<0.001	−5.37	0.04	
IQCG	4.48	0.012	1.07	0.6	In patients with allergic rhinitis
BBS1	−3.23	<0.001	−1.05	0.8	
RBM8A	−1.7	0.07	−3.01	0.039	In patients with allergic rhinitis with asthma
SSR1	−2.38	0.2	−3.74	0.044	
CLN8	−2.53	0.06	−5.34	0.028	

FC = fold change; *p*-value = *p*-value adjusted for multiple testing.

**Table 5:** Disease-specific genes induced in the lower airways

**Gene ID**	**FC rhinitis**	***p*-value rhinitis**	**FC asthma**	***p*-value asthma**	**Disease-specific**
CFB	42.13	0.001	30.04	0.009	Significant *p*-value in patients with allergic rhinitis with or without asthma
MB	8.95	0.005	2.73	0.017	
MAMDC2	3.2	0.009	1.58	0.06	
XRCC6BP1	−2.01	0.005	−3.06	0.01	
C4orf32	−3.06	0.027	−1.36	0.018	
MPPE1	−3.31	0.016	−3.83	0.026	
NEBL	−3.8	0.009	−3.35	0.012	
GPC6	−5.11	0.001	−2.31	0.022	
CYP1B1	−6.31	0.027	−2.39	0.027	
CLN8	−6.39	0.001	−5	0.011	
SHISA2	4.61	0.015	1.29	0.4	In patients with allergic rhinitis
S100A7A	4.07	0.017	1.38	0.4	
NPFFR2	3.7	0.043	1.26	0.2	
RDH10	3.37	0.016	1.1	0.6	
BMP6	3.36	0.015	1.11	0.6	
RNF141	−3.04	0.005	1.08	0.3	
C12orf28	−3.08	0.037	−1.14	0.4	
LOC148189	−3.23	0.023	−1.13	0.4	
CRNN	−3.75	0.021	−1.07	0.9	
PCK2	−4.16	0.024	−1.25	0.2	
CTH	−5.08	0.046	−1.23	0.4	
FLG	−5.17	0.004	−1.53	0.2	
FANCD2	1.03	0.2	−3.03	0.014	In patients with allergic rhinitis with asthma
MYLK	−1.03	0.5	−3.1	0.005	
SSR1	−1.16	0.4	−3.13	0.025	

FC = fold change; *p*-value = *p*-value adjusted for multiple testing.

## Discussion

This study shows the poly(I:C)-induced gene expression profiles of both upper and lower airway epithelium of patients with asthma, allergic rhinitis and healthy controls. The transcriptional response to poly(I:C) was characterized by a strong induction of genes. Among these genes were those involved in the response to virus, apoptotic processes and antigen presentation. Although the majority of genes involved in the response to poly(I:C) were similarly induced in upper and lower airways in all groups, we also observed differential expression, in particular with regard to impaired interferon expression in asthma. Furthermore, in contrast to healthy controls and rhinitis patients, the upper and lower airways of patients with asthma did not show poly(I:C)-induced down-regulation of mitochondrial genes. These findings are indicative of mitochondrial dysfunction in airway epithelium of patients with allergic asthma, and identify genes that may play a role in the altered viral response of diseased upper as well as lower airway epithelium.

To our knowledge, this is the first study that extensively profiles gene expression by microarray of the combined upper and lower airways epithelium in response to dsRNA of healthy individuals and patients with allergic rhinitis with or without allergic asthma. In our earlier study we describe the differences in gene expression profiles at baseline levels [11]. Interestingly, differential expression between the groups is mainly observed in both upper and lower airways, resulting in primarily comparable responses to dsRNA infection by the upper and lower airway epithelium within subjects. Furthermore, only small differences are observed between healthy controls and allergic rhinitis patients. Apparently, a pre-existing inflammatory background in allergic rhinitis does not grossly affect the response to poly(I:C). This suggests that the inflammatory pathways as induced by allergens and rhinovirus are mostly diverse.

Our finding of similar induction of genes involved in inflammatory responses in the airway epithelium in all three groups extends previous results. In a previous study examining the transcriptional response of RV-infected primary bronchial epithelial cells, pro-inflammatory pathways were similarly induced in asthma and controls [7]. Our data show that this also holds for allergic rhinitis. Hence, according to these results, the majority of inflammatory genes in epithelial cells are induced by viruses in both the healthy and diseased states. However, this can not be extrapolated to COPD, in which bronchial epithelial cells demonstrated enhanced pro-inflammatory and antiviral reactions to RV as compared to healthy controls [21].

Still, we also observed several interferon related genes that were induced in healthy controls and rhinitis patients, though not in asthma patients (especially the lower airways). Interferon-β1 (*IFNB1*) was significantly up-regulated in all cultures of all groups except for the lower airways of asthma patients. IL28B, known as interferon-λ3, was also significantly induced in both upper and lower airways of allergic rhinitis patients and controls, though not in asthma patients. This impaired induction in asthma was not due to high baseline gene expression prior to stimulation. IFN-βs and IFN-λs play a major role in the host defense against respiratory viral infections [8,22]. These results confirm and extend previous studies on RV-infected primary bronchial epithelial cells, demonstrating reduced IFN-β [9] and IFN-λ [8] response in asthma, suggesting a higher susceptibility to viral infections and thereby to exacerbations. This may partly explain the high correlation between viral respiratory tract infections and asthma exacerbations [1]. Nevertheless, when considering the most highly up-regulated genes, a considerable proportion of interferon-related genes appear to be induced in all groups. This fits in with very recent data in human bronchial smooth muscle cells, also showing production of IFNs by poly(I:C) [23]. Notably, the upregulation of these interferon-related genes tends to be much less in patients with asthma, although a larger fold change difference of a gene might not necessarily be linked to larger impact on a protein pathway.

The loss of mitochondrial and other metabolic gene down-regulation to dsRNA in asthma as compared to healthy controls and allergic rhinitis is in keeping with previously reported modifications in mitochondrial/metabolic function in A549 cells and animal models of allergic inflammation. These studies demonstrated that mitochondrial dysfunction exacerbates antigen-driven allergic airway inflammation by increased generation of reactive oxygen species (ROS), which induces oxidative stress in the lungs [24]. Environmental factors such as allergens, ozone, and viruses increase ROS production thereby interactively promoting allergic inflammation [25,26]. Second, since viral replication is dependent on host resources, down-regulation of mitochondrial function in a healthy state may prevent energy production to provide this replication. A recent review describes previous studies that have observed modulation of mitochondrial functions during different viral infections [27]. That viral replication depends on mitochondrial biogenesis was previously observed during Human Cytomegalovirus infection [28]. Interestingly, this loss of down-regulated genes in our study was observed in both upper and lower airways of patients with asthma, suggesting changed host characteristics of the upper airways in patients with allergic rhinitis plus asthma as compared to those with allergic rhinitis alone. This implies at least an interaction between asthma and rhinitis in the response to respiratory viruses. Since we did not include asthma patients without allergic rhinitis we can only speculate about the role of the presence of allergic rhinitis in asthma. However, in our previous study we found a large impact of allergic rhinitis on the differences in epithelial gene expression between upper and lower airways, influencing the lower airways as well [11]. Therefore, we assume that allergic rhinitis affects both upper and lower epithelial responses to viruses in patients with asthma. This would further explain mainly similar responses to dsRNA by both nasal and bronchial epithelium within subject-groups.

Among the disease-specific genes that were induced in allergic rhinitis patients with or without asthma but not in healthy controls, there were genes (*ERAP1, LAMP2*) that have been shown to be involved in antigen presentation [29,30]. Furthermore, several genes (*SH3KBP1, CRNN, FLG, S100A7A*) related to allergic inflammation [31-34] were either induced in allergic rhinitis patients with or without asthma (*SH3KB1)* or in allergic rhinitis patients only (*CRNN, FLG, S100A7A*). The genes *BMP6, CFB and MYLK* have previously been associated with airway inflammation and hyperresponsiveness [35-37]. Of these genes, *CFB* was induced in all patients, whereas *BMP6* solely in allergic rhinitis patients and *MYLK* in allergic rhinitis patients with asthma. The *AHNAK* gene, induced in allergic rhinitis patients with or without asthma, was previously associated with asthma susceptibility [38]. Interestingly, there was also induction of ciliary genes (*BBS1, NEBL,* NME7) [39-41] of which *NEBL* and *NME7* in allergic rhinitis patients with or without asthma while *BBS1* exclusively in patients with allergic rhinitis. Mucociliary clearance is essential for the pulmonary defense [42], and ciliary dysfunction has been previously related to asthma severity [43].

The strength of our study is that we have collected primary epithelial cells from both upper and lower airways from the same individuals in three different conditions (healthy, allergic rhinitis, allergic rhinitis and asthma). Furthermore, we were able to extensively analyse gene expression by using microarray, which was confirmed by PCR analysis. Nevertheless, the study has some limitations. Firstly, we included relatively few individuals per group. We carefully calculated this samples size by setting the false-discovery rate at approximately 5%. A larger sample size would have allowed capturing smaller differences in expression profile or greater differences in genes that display a large variation in expression per individual. As a consequence we purposely focused on paired differences before and after poly(I:C) stimulation instead of comparing unpaired expression between the different subject groups. In addition to the original power calculation mentioned, the statistics used to measure differential expression applied the Benjamini and Hochberg adjustment of p-values for multiple testing. This correction uses a smaller significance level, inevitably reducing the power of the analysis. This will have led to false-negative results on poly(I:C)-induced genes, but it purposely limited the risk of false positive discovery.

We did not use a real virus as a stimulus, but we used poly(I:C) instead. Although a real virus such as RV would have been preferable, in order to match the *in vivo* conditions, dsRNA is an adequate surrogate marker for RV. Toll-like receptor 3 (TLR3) is required for the sensing of RV produced dsRNA [10] and the TLR3 ligand poly(I:C) was found previously to be a very effective stimulus of airway epithelial cells [44,45]. Since actual steroid exposure will change the expression of many genes, patients were prohibited steroid usage for 4 weeks before sampling. In fact, all allergic rhinitis patients were entirely steroid naïve and only one asthma patient used topical steroids and one asthma patient used inhaled steroids 4 weeks prior to recruitment. We cannot exclude carry-over effects of steroids even over this time span. Finally, culturing of cells will affect gene expression levels since conditions are no longer the same as in the airways. This seems to be inevitable. Alternative procedures to obtain epithelial cells such as laser capture or direct measurement after isolation might mitigate these effects of cell culturing, but will introduce new biases introduced by contamination by other cell types and/or the (enzymatic) isolation procedures themselves. However, as we compared epithelial cells from the same individuals, the standardized culturing of these cells should have affected nose and bronchial epithelial cells similarly.

The currently observed disease-related differences in viral-induced gene expression of upper and lower airways between patients with allergic rhinitis and asthma and healthy controls may have clinical implications. First, these results help to understand the mechanistic pathways of the mutual interaction between asthma and rhinitis, for which there is considerable clinical evidence [46]. Second, the current analysis identified several new genes whilst confirming other genes from previous findings. This may provide potential targets with respect to mitochondrial dysfunction and interferon involved genes for drug-discovery studies and for treatment of exacerbations in patients with combined upper and lower airway disease.

## Conclusions

In conclusion, we demonstrated that there are differences between rhinitis patients with and without asthma in the epithelial expression of dsRNA-induced genes, which are related to interferons and mitochondrial function. This appears to be manifested in both the upper and lower airways, suggesting mainly comparable responses to dsRNA infection in upper and lower airway epithelium within subjects. These data identify host-virus interactions in asthma, rhinitis and controls, which is required for developing targeted preventative and therapeutic interventions in asthma exacerbations.

## Abbreviations

ARIA: Allergic rhinitis and its impact on asthma; BEBM: Bronchial epithelial basal medium; dsRNA: Double-stranded RNA; FC: Fold change; GINA: Global initiative for asthma; GO: Gene ontology; PCR: Polymerase chain reaction; RV: Rhinovirus.

## Competing interests

All authors declare they have no competing interests

## Authors’ contributions

AHW, WJF, EHB, PJS, CMD participated in the study’s conception and design. AHW recruited the subjects. AHW and SL performed the experiments. AHW and AHZ performed the statistical analysis and all authors contributed in the interpretation of data, preparation and editing of the manuscript for intellectual content. All authors read and approved the final manuscript.

## Supplementary Material

Additional file 1 **Table S1.** Validatory PCR of housekeeping genes and significantly different genes. **Table S2.** dsRNA-induced genes in upper and lower airways. **Table S3A.** Genes assigned to GO-cluster response to virus induced in the upper airways. **Table S3B.** Genes assigned to GO-cluster esponse to virus induced in the lower airways **Table S4.** Genes induced in the upper airways of healthy controls and allergic rhinitis patients, assigned to GO cluster Mitochondrion. **Table S5.** Genes induced in the lower airways of healthy controls, assigned to GO cluster Mitochondrion. **Table S6.** Genes induced in the lower airways of healthy controls and allergic rhinitis patients, assigned to GO cluster Mitochondrion. Click here for file

Additional file 2: Figure S1 Correlation plot of real-time PCR data and microarray results. FCs were logtranformed. Click here for file
