# Supplementary material for: dsRNA-induced changes in gene expression profiles of primary nasal and bronchial epithelial cells from patients with asthma, rhinitis and controls
Source: Respir Res. 2014 Jan 29;15(1):9. doi: 10.1186/1465-9921-15-9 (PMC3916078; doi:10.1186/1465-9921-15-9)
Supplement: Additional file 1 — Table S1. Validatory PCR of housekeeping genes and significantly different genes. Table S2. dsRNA-induced genes in upper and lower airways. Table S3A. Genes assigned to GO-cluster response to virus induced in the upper airways. Table S3B. Genes assigned to GO-cluster esponse to virus induced in the lower airways Table S4. Genes induced in the upper airways of healthy controls and allergic rhinitis patients, assigned to GO cluster Mitochondrion. Table S5. Genes induced in the lower airways of healthy controls, assigned to GO cluster Mitochondrion. Table S6. Genes induced in the lower airways of healthy controls and allergic rhinitis patients, assigned to GO cluster Mitochondrion. [file 1465-9921-15-9-S1.doc]

**ONLINE SUPPLEMENT**

TITLE PAGE

Original Article

**dsRNA-induced changes in gene expression profiles of primary nasal and bronchial epithelial cells from patients with asthma, rhinitis and controls**

Ariane H Wagener, MD,^a^ Aeilko H Zwinderman, PhD,^b^ Silvia Luiten,^c^ Wytske J Fokkens, MD, PhD,^c^ Elisabeth H Bel, MD, PhD,^a^ Peter J Sterk, MD, PhD,^a^ and Cornelis M van Drunen, PhD,^c^

^a^Department of Respiratory Medicine, Academic Medical Center, University of Amsterdam, The Netherlands

^b^Department of Clinical Epidemiology, Biostatistics & Bioinformatics, Academic Medical Center, University of Amsterdam, The Netherlands

^c^Department of Otorhinolaryngology, Academic Medical Center, University of Amsterdam, The Netherlands

**Corresponding author**

Ariane H. Wagener, MD

Department of Respiratory Medicine, F5-260

Academic Medical Center (AMC)

University of Amsterdam

Meibergdreef 9

1105 AZ Amsterdam

The Netherlands

Telephone: +31 (0)20 5661660

E-mail: a.h.wagener@amc.uva.nl

**METHODS**

**Primary epithelial cell culture**

Epithelal cell cultures were done as previously described [1]. Primary cells were obtained by first digesting the biopsies and brushes with collagenase 4 (Worthington Biochemical Corp., Lakewood, NJ, USA) for 1 hour in Hanks’ balanced salt solution (Sigma-Aldrich, Zwijndrecht, The Netherlands). Subsequently cells were washed with Hanks’ balanced salt solution (HBSS) and resuspended in BEGM (Invitrogen, Breda, The Netherlands) and seeded in one well of a 6 wells plate. Cells were grown in fully humidified air containing 5% CO_2_ at 37°C, and culture medium was replaced every other day. Cells were cultured to 80% confluence and were pre-incubated with BEBM for 24 hours prior to exposure to BEBM containing 20µg/ml poly(I:C) or with BEBM alone (control condition) for 24 hours before removal of supernatant and RNA extraction. For bronchial epithelial cells it took 14 days on average, and for nasal epithelial cells it took 24 days on average to grow to 80% confluence. There was no difference in time of culture between the three subject groups.

**RNA extraction**

Total RNA from each sample was extracted using Trizol (Life Technologies Inc., Gaitersburg, MD, USA) using manufacturer’s protocol, followed by purification by nucleospin RNA II (Machery-Nagel, Düren, Germany). RNA concentration of all samples was measured on the nanodrop ND-1000 (NanoDrop Technologies Inc., Wilmington, DE, USA). The quality of the RNA was checked by using Agilent 2100 bio-analyser (Agilent Technologies, Palo Alto, CA, USA). All RIN scores were ≥9.5.

**Microarray Affymetrix U133+ PM**

Microarray analysis was done by previously published method [1]. Human Genome U133+ PM Genechip Array (Affymetrix inc., Santa Clara, CA, USA) representing more than 47,000 transcripts and variants, including over 33,000 well-characterized genes, was used in the analysis of the genes. The MicroArray Department (MAD) of the University of Amsterdam, a fully licensed microarray technologies centre for Affymetrix Genechip® platforms, performed the technical handling and the quality control of the microarray experiments. The quality of the images was checked by visual inspection and all raw data passed quality criteria based on borderplots, pseudocolor slide images, RNA degradation plots, box and density plots, RI plots (against a pseudoreference), correlation and PCA plots.

**Real-time polymerase chain reaction and analysis**

Quantitative real-time PCR was used to validate the differential expression of selected genes. A random selection of 9 genes was used that showed a variance of responses to Poly(I:C). PCR was performed on Bio-Rad CFX96 real-time PCR detection system (Bio-Rad, Veenendaal, The Netherlands). SYBR® Green primer sequences for IL13-Rα2, EREG, PDE4D, IL1-β, IP-10, TIMP2, IL8, β –actin and GAPDH were obtained from Sigma-Aldrich (Sigma-Aldrich, Zwijndrecht, The Netherlands). The following primers were used: IL13-Rα2; sense: TGC-TCA-GAT-GAC-GGA-ATT-TGG, antisense: TGG-TAG-CCA-GAA-ACG-TAG-CAA-AG, EREG; sense: ATC-CTG-GCA-TGT-GCT-AGG-GT, antisense: GTG-CTC-CAG-AGG-TCA-GCC-AT, PDE4D; sense: GGC-CTC-CAA-CAA-GTT-TAA-AA, antisense: ACC-AGA-CAA-CTC-TGC-TAT-TCT, IL1-β; sense: GGA-TAT-GGA-GCA-ACA-AGT-GG, antisense: ATG-TAC-CAG-TTG-GGG-AAC-TG, IP-10; sense: TGA-AAT-TAT-TCC-TGC-AAG-CCA-AT, antisense: CAG-ACA-TCT-CTT-CTC-ACC-CTT-CTT-T, TIMP2; sense: ATA-AGC-AGG-CCT-CCA-ACG-C, antisense: GAG-CTG-GAC-CAG-TCG-AAA-CC, IL8; sense: CCA-CAC-TGC-GCC-AAC-ACA-GAA-ATT-ATT-G, antisense: GCC-CTC-TTC-AAA-AAC-TTC-TCC-ACA-ACC-C, β –actin; sense: TGA-GCG-CGG-CTA-CAG-CTT, antisense: TCC-TTA-ATG-TCA-CGC-ACG-ATT-T, GAPDH; sense: GAA-GGT-GAA-GGT-CGG-AGT-C, antisense: GAA-GAT-GGT-GAT-GGG-ATT-TC. For ATF3 and DUSP1 we used TaqMan® gene expression assays from Applied Biosystems (Nieuwerkerk a/d IJssel, The Netherlands) with the following assay IDs: ATF3; HS00231069_M1, DUSP1; HS006102757_G1.

Correlations between fold changes (FC) within the microarray data and the real-time PCR data were determined using Pearson’s correlation.

**RESULTS**

**Validation of microarray data**

The results of this microarray experiment were validated by independent real time PCR on the same starting material used for the microarray analysis. We first determined the expression of the housekeeping genes (ACTB and GAPDH) which were not statistically significantly induced by poly(I:C). Table S1 shows the fold changes (FC) calculated from the microarray data and the real-time PCR-derived expression. Statistical analysis revealed a high level of correspondence (R=0.929, P<0.0001) between the microarray data and the real-time PCR (Figure S1). FCs were logtranformed because of such large variances.

**REFERENCES**

1. Wagener AH, Zwinderman AH, Luiten S, Fokkens WJ, Bel EH, Sterk PJ, van Drunen CM. **The impact of allergic rhinitis and asthma on human nasal and bronchial epithelial gene expression**. *PLoS One*, in press.

**Table S1.** Validatory PCR of housekeeping genes and significantly different genes.

|  | **Bronchial epithelial cells** | | | | | |
| --- | --- | --- | --- | --- | --- | --- |
|  | Healthy | | Allergic rhinitis | | Allergic rhinitis & asthma | |
|  | PCR FC | Microarray FC | PCR FC | Microarray FC | PCR FC | Microarray FC |
| ACTB | 1.16 | -1.03 | 1.52 | 1.04 | 1.38 | -1.11 |
| GAPDH | -1.83 | -1.00 | -1.82 | -1.03 | -1.49 | -1.03 |
| ATF3 | 1.43 | 2.37 | -1.78 | 1.21 | -2.10 | 1.51 |
| CXCL10 | 568.10 | 263.55 | 214.38 | 121.34 | 170.31 | 88.92 |
| DUSP1 | 5.78 | 6.71 | 4.01 | 2.35 | 4.96 | 4.16 |
| EREG | 1.37 | 2.07 | -3.59 | -1.83 | -1.26 | 1.17 |
| IL13RA2 | 10.59 | 14.62 | 7.32 | 8.66 | 7.41 | 13.44 |
| IL1B | 2.83 | 3.52 | 1.30 | 1.75 | 2.43 | 3.11 |
| IL8 | 32.48 | 13.98 | 44.45 | 23.40 | 26.06 | 16.60 |
| PDE4D | -3.63 | -1.33 | -6.19 | -1.15 | -3.81 | -1.33 |
| TIMP2 | 1.50 | 2.67 | 1.29 | 2.39 | 1.52 | 2.56 |

|  | **Nasal epithelial cells** | | | | | |
| --- | --- | --- | --- | --- | --- | --- |
|  | Healthy | | Allergic rhinitis | | Allergic rhinitis & asthma | |
|  | PCR FC | Microarray FC | PCR FC | Microarray FC | PCR FC | Microarray FC |
| ACTB | -1.43 | -1.04 | 1.04 | -1.05 | -1.03 | -1.06 |
| GAPDH | -1.92 | -1.04 | -2.45 | -1.06 | -1.77 | -1.02 |
| ATF3 | 9.61 | 5.02 | 4.35 | 3.97 | 1.69 | 2.75 |
| CXCL10 | 465.72 | 74.79 | 587.32 | 175.30 | 161.27 | 72.35 |
| DUSP1 | 11.45 | 17.60 | 7.48 | 15.22 | 5.98 | 7.17 |
| EREG | 3.90 | 3.13 | 1.67 | 3.24 | 1.03 | 1.35 |
| IL13RA2 | 22.16 | 12.13 | 17.20 | 14.39 | 6.15 | 5.83 |
| IL1B | 5.25 | 3.33 | 3.02 | 4.04 | 4.14 | 3.75 |
| IL8 | 178.73 | 73.41 | 122.11 | 64.77 | 63.85 | 23.72 |
| PDE4D | -1.09 | -1.03 | -7.87 | -1.29 | -1.86 | -1.04 |
| TIMP2 | 3.02 | 4.25 | 1.77 | 5.73 | 1.31 | 2.97 |

PCR expression is given as fold change (FC).

**Table S2.** dsRNA-induced genes in upper and lower airways

|  | Healthy | | Healthy | | Rhinitis | | Rhinitis | | Asthma | | Asthma | |
| --- | --- | --- | --- | --- | --- | --- | --- | --- | --- | --- | --- | --- |
|  | Nose | | Bronchus | | Nose | | Bronchus | | Nose | | Bronchus | |
| Probesets  *P*<0.05 | 17402 | | 13424 | | 15538 | | 7517 | | 6996 | | 8560 | |
| Probesets  *P*<0.05 &FC>4 | 1255 | | 550 | | 1439 | | 517 | | 517 | | 427 | |
| Genes  *P*<0.05 | 10163 | ↑4636  ↓5527 | 8342 | ↑3638  ↓4704 | 9353 | ↑4204  ↓5149 | 5190 | ↑2294  ↓2896 | 4919 | ↑2293  ↓2626 | 5810 | ↑2621  ↓3189 |
| Genes  *P*<0.05 & FC>4 | 894 | ↑528  ↓366 | 401 | ↑274  ↓127 | 1037 | ↑586  ↓451 | 391 | ↑244  ↓147 | 383 | ↑275  ↓108 | 319 | ↑227  ↓92 |

FC=fold change; *P*=*p*-value adjusted for multiple testing

**Table S3A. Genes assigned to GO-cluster *response to virus* induced in the upper airways**

| Gene symbol | Venn diagram | FC Healthy | FC Rhinitis | FC Asthma |
| --- | --- | --- | --- | --- |
| C7orf25 /// PSMA2 | A | 1.30 |  |  |
| CFL1 | A | -1.17 |  |  |
| LILRB1 | A | 1.21 |  |  |
| PIM2 | A | -1.32 |  |  |
| POLR3F | A | -1.22 |  |  |
| ACTA2 | B | -1.27 | -1.34 |  |
| BANF1 | B | -1.56 | -1.59 |  |
| BECN1 | B | -1.25 | -1.25 |  |
| BNIP3L | B | 1.53 | 1.76 |  |
| CCL22 | B | 1.42 | 1.33 |  |
| CREBZF | B | -1.57 | -1.53 |  |
| CYP1A1 | B | -2.60 | -1.73 |  |
| DNAJC3 | B | 1.95 | 1.81 |  |
| EEF1G /// TUT1 | B | -1.77 | -1.85 |  |
| ENO1 | B | -1.33 | -1.38 |  |
| FOSL1 | B | 3.39 | 3.90 |  |
| HBXIP | B | -1.23 | -1.32 |  |
| IFNAR1 | B | 1.53 | 1.39 |  |
| IL12A | B | 2.64 | 2.96 |  |
| IL28B | B | 4.53 | 7.30 |  |
| LYST | B | 1.99 | 1.73 |  |
| MAVS | B | -2.00 | -1.69 |  |
| POLR3A | B | 1.53 | 1.65 |  |
| POLR3H | B | -2.61 | -2.44 |  |
| POLR3K | B | -2.95 | -2.52 |  |
| TBK1 | B | 1.83 | 1.49 |  |
| ABCE1 | C | -3.91 | -4.42 | -2.48 |
| ACE2 | C | 15.24 | 12.25 | 7.55 |
| AP1S1 | C | -1.73 | -1.63 | -1.40 |
| APOBEC3F | C | 2.78 | 2.80 | 2.29 |
| APOBEC3G | C | 11.38 | 10.82 | 5.06 |
| BCL2 | C | -1.24 | -1.40 | -1.46 |
| BCL3 | C | 3.08 | 3.17 | 2.43 |
| BNIP3 | C | 2.05 | 2.05 | 1.66 |
| BST2 | C | 45.73 | 79.99 | 27.17 |
| C19orf2 | C | -1.60 | -1.90 | -1.43 |
| CCDC130 | C | 1.40 | 1.41 | 1.27 |
| CCL4 | C | 255.13 | 243.83 | 39.32 |
| CCL5 | C | 517.58 | 717.90 | 148.81 |
| CCT5 | C | -3.44 | -3.23 | -2.21 |
| DDX58 | C | 25.21 | 38.03 | 14.32 |
| DUOX2 | C | 3.01 | 3.86 | 2.03 |
| EIF2AK2 | C | 4.09 | 6.12 | 3.56 |
| GPAM | C | -4.25 | -4.77 | -2.70 |
| GTF2F1 | C | 1.31 | 1.55 | 1.33 |
| HERC5 | C | 46.50 | 72.10 | 26.64 |
| HNRNPUL1 | C | -1.35 | -1.37 | -1.27 |
| HSPB1 | C | -1.99 | -2.25 | -1.80 |
| IFI16 | C | 1.70 | 1.59 | 1.74 |
| IFI35 | C | 38.88 | 67.89 | 27.76 |
| IFI44 | C | 64.88 | 164.77 | 42.51 |
| IFIH1 | C | 18.03 | 22.18 | 10.42 |
| IFITM1 | C | 8.15 | 10.90 | 6.25 |
| IFITM2 | C | 2.92 | 2.99 | 2.45 |
| IFITM3 | C | 2.27 | 2.25 | 1.93 |
| IFNAR2 | C | 3.40 | 3.13 | 2.38 |
| IFNB1 | C | 3.17 | 2.77 | 1.77 |
| IFNE | C | -1.88 | -2.68 | -2.45 |
| IFNGR1 | C | 1.82 | 1.48 | 1.68 |
| IFNGR2 | C | 2.45 | 2.36 | 1.99 |
| IFNK | C | 3.23 | 4.05 | 3.67 |
| IL23A | C | 20.52 | 24.06 | 6.43 |
| IL28A | C | 13.29 | 15.78 | 4.49 |
| IL29 | C | 25.01 | 20.48 | 7.40 |
| IL6 | C | 20.34 | 11.13 | 10.02 |
| IRF3 | C | 1.57 | 1.56 | 1.40 |
| IRF7 | C | 28.26 | 40.73 | 16.88 |
| IRF9 | C | 2.87 | 3.41 | 2.94 |
| ISG15 | C | 23.50 | 46.89 | 15.63 |
| ISG20 | C | 32.65 | 41.94 | 14.82 |
| ITCH | C | 2.67 | 3.02 | 2.43 |
| IVNS1ABP | C | -5.44 | -6.14 | -2.57 |
| MST1R | C | 2.95 | 3.39 | 2.04 |
| MX1 | C | 30.17 | 92.06 | 21.00 |
| MX2 | C | 115.45 | 287.26 | 72.32 |
| MYD88 | C | 2.60 | 2.98 | 2.37 |
| NLRC5 | C | 38.61 | 38.77 | 12.56 |
| ODC1 | C | -2.23 | -1.78 | -2.04 |
| PCBP2 | C | -1.49 | -1.56 | -1.38 |
| PLSCR1 | C | 6.38 | 8.28 | 5.22 |
| POLR3B | C | -1.44 | -1.63 | -1.45 |
| POLR3C | C | 1.42 | 1.30 | 1.27 |
| POLR3D | C | 1.70 | 1.91 | 1.55 |
| POLR3E | C | -2.14 | -2.32 | -1.67 |
| POLR3G | C | -1.87 | -2.15 | -1.36 |
| PRKRA | C | -1.90 | -2.11 | -1.61 |
| PVR | C | 1.93 | 1.81 | 1.54 |
| RELA | C | 2.38 | 2.45 | 1.98 |
| RPS15A | C | -8.15 | -9.98 | -4.19 |
| RSAD2 | C | 260.58 | 638.34 | 98.63 |
| SAMHD1 | C | 16.36 | 31.76 | 12.09 |
| SIVA1 | C | -2.16 | -2.48 | -1.57 |
| STAT1 | C | 13.66 | 17.43 | 10.47 |
| STAT2 | C | 3.50 | 4.78 | 3.40 |
| STMN1 | C | -5.68 | -4.89 | -2.96 |
| TICAM1 | C | 3.14 | 3.12 | 2.38 |
| TLR3 | C | 11.82 | 10.00 | 6.68 |
| TNF | C | 8.16 | 6.72 | 4.38 |
| TRIM11 | C | 1.73 | 1.78 | 1.59 |
| TRIM22 | C | 4.90 | 5.16 | 4.61 |
| TRIM25 | C | 6.43 | 8.67 | 4.13 |
| TRIM5 | C | 3.81 | 3.90 | 2.94 |
| UNC13D | C | 1.60 | 1.76 | 1.53 |
| UNC93B1 | C | 2.40 | 3.27 | 2.33 |
| XPO1 | C | -1.51 | -1.63 | -1.41 |
| XPR1 | C | -1.76 | -2.49 | -1.23 |
| ZC3HAV1 | C | 16.13 | 18.83 | 9.78 |
| ZNF175 | C | 1.67 | 1.80 | 1.32 |
| ABCC9 | E |  | 1.23 |  |
| CLU | E |  | -1.26 |  |
| DMBT1 | E |  | 1.44 |  |
| MICA /// MICB | E |  | 1.21 |  |
| PSMA2 | E |  | -1.27 |  |
| PTPRC | E |  | 1.24 |  |
| IRAK3 | F |  | -1.82 | 1.38 |
| CDK6 | G |  |  | -1.41 |

**Venn diagram**: **A**=healthy specific; **B**=overlap healthy–rhinitis; **C**=overlap all groups; **D**=overlap healthy–asthma; **E**=rhinitis specific; **F**=overlap rhinitis–asthma; **G**=asthma specific. **FC** = fold change.

**Table S3B. Genes assigned to GO-cluster *response to virus* induced in the lower airways**

| Gene symbol | Venn diagram | FC Healthy | FC Rhinitis | FC Asthma |
| --- | --- | --- | --- | --- |
| BANF1 | A | -1.36 |  |  |
| BECN1 | A | -1.23 |  |  |
| BNIP3L | A | 1.30 |  |  |
| CXCL12 | A | -1.20 |  |  |
| ENO1 | A | -1.21 |  |  |
| IRF3 | A | 1.31 |  |  |
| MAVS | A | -1.47 |  |  |
| MST1R | A | 1.37 |  |  |
| POLR3D | A | 1.40 |  |  |
| POLR3G | A | -1.62 |  |  |
| PSMA2 | A | -1.30 |  |  |
| RNASEL | A | -1.35 |  |  |
| SIVA1 | A | -1.73 |  |  |
| XPR1 | A | -1.45 |  |  |
| BNIP3 | B | 1.55 | 1.69 |  |
| IFNAR1 | B | 1.23 | 1.42 |  |
| IFNB1 | B | 1.60 | 1.47 |  |
| IFNGR1 | B | 1.64 | 1.50 |  |
| IL28B | B | 2.40 | 2.16 |  |
| LYST | B | 1.73 | 1.68 |  |
| POLR3E | B | -1.62 | -1.93 |  |
| POLR3K | B | -2.11 | -2.00 |  |
| ZNF175 | B | 1.54 | 1.88 |  |
| ABCE1 | C | -2.06 | -2.49 | -1.89 |
| APOBEC3F | C | 1.90 | 1.76 | 2.11 |
| APOBEC3G | C | 6.62 | 5.28 | 8.11 |
| BCL3 | C | 2.44 | 2.45 | 1.98 |
| BST2 | C | 24.79 | 48.02 | 13.00 |
| C19orf2 | C | -1.42 | -1.56 | -1.43 |
| CCL4 | C | 83.19 | 20.90 | 35.57 |
| CCL5 | C | 736.54 | 322.77 | 231.87 |
| CCT5 | C | -2.10 | -1.80 | -1.85 |
| CDK6 | C | -1.46 | -1.30 | -1.52 |
| CREBZF | C | -1.29 | -1.36 | -1.38 |
| CYP1A1 | C | -1.91 | -2.85 | -2.10 |
| DDX58 | C | 28.49 | 23.14 | 18.03 |
| DNAJC3 | C | 1.48 | 1.36 | 1.40 |
| DUOX2 | C | 9.56 | 4.80 | 7.87 |
| EEF1G /// TUT1 | C | -1.53 | -1.57 | -1.38 |
| EIF2AK2 | C | 4.63 | 5.40 | 3.60 |
| GPAM | C | -2.90 | -2.26 | -2.44 |
| HERC5 | C | 35.87 | 25.29 | 27.95 |
| HNRNPUL1 | C | -1.26 | -1.27 | -1.25 |
| HSPB1 | C | -1.53 | -1.74 | -1.61 |
| IFI35 | C | 48.10 | 39.73 | 31.80 |
| IFI44 | C | 236.92 | 70.93 | 104.82 |
| IFIH1 | C | 18.21 | 6.27 | 12.76 |
| IFITM1 | C | 14.95 | 10.61 | 11.08 |
| IFITM2 | C | 3.33 | 3.65 | 3.48 |
| IFITM3 | C | 2.97 | 3.53 | 2.73 |
| IFNAR2 | C | 2.43 | 2.54 | 2.36 |
| IFNGR2 | C | 2.25 | 1.87 | 2.01 |
| IL23A | C | 9.34 | 12.37 | 7.90 |
| IL28A | C | 4.21 | 4.48 | 4.99 |
| IL29 | C | 6.80 | 3.95 | 4.96 |
| IL6 | C | 8.80 | 4.92 | 7.60 |
| IRF7 | C | 26.30 | 19.88 | 20.32 |
| IRF9 | C | 4.44 | 3.44 | 3.67 |
| ISG15 | C | 49.88 | 64.58 | 32.63 |
| ISG20 | C | 10.51 | 7.46 | 8.46 |
| ITCH | C | 1.93 | 1.75 | 1.86 |
| IVNS1ABP | C | -2.43 | -2.16 | -2.07 |
| MX1 | C | 140.44 | 175.11 | 67.81 |
| MX2 | C | 201.46 | 163.72 | 96.81 |
| MYD88 | C | 2.17 | 2.32 | 2.19 |
| NLRC5 | C | 17.88 | 12.59 | 11.61 |
| ODC1 | C | -2.02 | -2.87 | -2.11 |
| PCBP2 | C | -1.29 | -1.29 | -1.31 |
| PLSCR1 | C | 7.77 | 7.54 | 5.70 |
| POLR3A | C | 1.49 | 1.61 | 1.29 |
| PRKRA | C | -1.59 | -1.41 | -1.57 |
| RELA | C | 1.91 | 1.62 | 1.69 |
| RPS15A | C | -4.45 | -4.65 | -3.17 |
| RSAD2 | C | 363.02 | 304.55 | 167.47 |
| SAMHD1 | C | 17.56 | 3.38 | 13.59 |
| STAT1 | C | 16.34 | 6.17 | 11.13 |
| STAT2 | C | 3.40 | 2.97 | 2.59 |
| TBK1 | C | 1.44 | 1.42 | 1.30 |
| TICAM1 | C | 2.30 | 2.15 | 2.31 |
| TLR3 | C | 6.70 | 3.71 | 5.54 |
| TNF | C | 4.81 | 3.95 | 4.35 |
| TRIM22 | C | 6.91 | 9.76 | 4.63 |
| TRIM25 | C | 3.96 | 3.13 | 3.72 |
| TRIM5 | C | 2.60 | 2.55 | 2.51 |
| UNC13D | C | 1.50 | 1.43 | 1.45 |
| UNC93B1 | C | 2.52 | 1.82 | 2.21 |
| ZC3HAV1 | C | 12.00 | 15.83 | 8.50 |
| ACE2 | D | 6.49 |  | 5.12 |
| ACTA2 | D | -1.36 |  | -1.38 |
| AP1S1 | D | -1.46 |  | -1.34 |
| BCL2 | D | -1.43 |  | -1.42 |
| CCDC130 | D | 1.26 |  | 1.23 |
| FOSL1 | D | 2.40 |  | 1.89 |
| GTF2F1 | D | 1.25 |  | 1.29 |
| IFI16 | D | 1.50 |  | 1.62 |
| IRAK3 | D | 1.40 |  | 1.56 |
| NLRP3 | D | 1.77 |  | 1.46 |
| POLR3H | D | -1.74 |  | -1.45 |
| PVR | D | 1.65 |  | 1.60 |
| STMN1 | D | -2.24 |  | -1.93 |
| TRIM11 | D | 1.59 |  | 1.54 |
| XPO1 | D | -1.25 |  | -1.31 |
| IFNE | E |  | -2.22 |  |
| CLU | G |  |  | -1.35 |

**Venn diagram**: **A**=healthy specific; **B**=overlap healthy–rhinitis; **C**=overlap all groups; **D**=overlap healthy–asthma; **E**=rhinitis specific; **F**=overlap rhinitis–asthma; **G**=asthma specific. **FC** = fold change.

**Table S4. Genes induced in the upper airways of healthy controls and allergic rhinitis patients, assigned to GO cluster *Mitochondrion*.**

| **Gene alias** | **Gene name/description** |
| --- | --- |
|  |  |
| AARS2 | alanyl-tRNA synthetase 2, mitochondrial (putative) |
| ABCA12 | ATP-binding cassette, sub-family A (ABC1), member 12 |
| ABCF2 | ATP-binding cassette, sub-family F (GCN20), member 2 |
| ACACB | acetyl-CoA carboxylase beta |
| ACAD8 | acyl-CoA dehydrogenase family, member 8 |
| ACAD9 | acyl-CoA dehydrogenase family, member 9 |
| ACADM | acyl-CoA dehydrogenase, C-4 to C-12 straight chain |
| ACADVL | acyl-CoA dehydrogenase, very long chain |
| ACN9 | ACN9 homolog (S. cerevisiae) |
| ACO2 | aconitase 2, mitochondrial |
| ACOT13 | acyl-CoA thioesterase 13 |
| ACP6 | acid phosphatase 6, lysophosphatidic |
| ACSF2 | acyl-CoA synthetase family member 2 |
| ACSL1 | acyl-CoA synthetase long-chain family member 1 |
| ACSS1 | acyl-CoA synthetase short-chain family member 1 |
| ADCK4 | aarF domain containing kinase 4 |
| ADH5 | alcohol dehydrogenase 5 (class III), chi polypeptide |
| ADO | 2-aminoethanethiol (cysteamine) dioxygenase |
| AIFM1 | apoptosis-inducing factor, mitochondrion-associated, 1 |
| AK2 | adenylate kinase 2 |
| AK3 | adenylate kinase 3 |
| AKAP10 | A kinase (PRKA) anchor protein 10 |
| AKT2 | v-akt murine thymoma viral oncogene homolog 2 |
| ALDH1B1 | aldehyde dehydrogenase 1 family, member B1 |
| ALDH1L1 | aldehyde dehydrogenase 1 family, member L1 |
| ALDH1L2 | aldehyde dehydrogenase 1 family, member L2 |
| ALDH2 | aldehyde dehydrogenase 2 family (mitochondrial) |
| ALDH5A1 | aldehyde dehydrogenase 5 family, member A1 |
| ALKBH1 | alkB, alkylation repair homolog 1 (E. coli) |
| ALKBH7 | alkB, alkylation repair homolog 7 (E. coli) |
| AP2M1 | adaptor-related protein complex 2, mu 1 subunit |
| APOA1BP | apolipoprotein A-I binding protein |
| ARG2 | arginase, type II |
| ARMC10 | armadillo repeat containing 10 |
| ATIC | 5-aminoimidazole-4-carboxamide ribonucleotide formyltransferase/IMP cyclohydrolase |
| ATP5B | ATP synthase, H+ transporting, mitochondrial F1 complex, beta polypeptide |
| ATP5C1 | ATP synthase, H+ transporting, mitochondrial F1 complex, gamma polypeptide 1 |
| ATP5D | ATP synthase, H+ transporting, mitochondrial F1 complex, delta subunit |
| ATP5G1 | ATP synthase, H+ transporting, mitochondrial F0 complex, subunit C1 (subunit 9) |
| ATP5G2 | ATP synthase, H+ transporting, mitochondrial F0 complex, subunit C2 (subunit 9) |
| ATP5G3 | ATP synthase, H+ transporting, mitochondrial F0 complex, subunit C3 (subunit 9) |
| ATP5H | ATP synthase, H+ transporting, mitochondrial F0 complex, subunit d |
| ATP5I | ATP synthase, H+ transporting, mitochondrial F0 complex, subunit E |
| ATP5J | ATP synthase, H+ transporting, mitochondrial F0 complex, subunit F6 |
| ATP5J2 | ATP synthase, H+ transporting, mitochondrial F0 complex, subunit F2 |
| ATP5L | ATP synthase, H+ transporting, mitochondrial F0 complex, subunit G |
| ATP5S | ATP synthase, H+ transporting, mitochondrial F0 complex, subunit s (factor B) |
| ATP5SL | ATP5S-like |
| ATPAF1 | ATP synthase mitochondrial F1 complex assembly factor 1 |
| BAX | BCL2-associated X protein |
| BBOX1 | butyrobetaine (gamma), 2-oxoglutarate dioxygenase (gamma-butyrobetaine hydroxylase) 1 |
| BCL2L1 | BCL2-like 1 |
| BCS1L | BCS1-like (yeast) |
| BDH2 | 3-hydroxybutyrate dehydrogenase, type 2 |
| BNIP3L | BCL2/adenovirus E1B 19kDa interacting protein 3-like |
| BOLA1 | bolA homolog 1 (E. coli) |
| BPHL | biphenyl hydrolase-like (serine hydrolase) |
| BRP44 | brain protein 44 |
| C10orf2 | chromosome 10 open reading frame 2 |
| C10orf58 | chromosome 10 open reading frame 58 |
| C12orf10 | chromosome 12 open reading frame 10 |
| C12orf62 | chromosome 12 open reading frame 62 |
| C14orf156 | chromosome 14 open reading frame 156 |
| C14orf159 | chromosome 14 open reading frame 159 |
| C15orf62 | chromosome 15 open reading frame 62 |
| C17orf61 | chromosome 17 open reading frame 61 |
| C17orf90 | chromosome 17 open reading frame 90 |
| C18orf19 | chromosome 18 open reading frame 19 |
| C19orf70 | chromosome 19 open reading frame 70 |
| C1orf151 | chromosome 1 open reading frame 151 |
| C20orf24 | chromosome 20 open reading frame 24 |
| C21orf33 | chromosome 21 open reading frame 33 |
| C2orf47 | chromosome 2 open reading frame 47 |
| C2orf64 | chromosome 2 open reading frame 64 |
| C3orf1 | chromosome 3 open reading frame 1 |
| C3orf31 | Chromosome 3 open reading frame 31 |
| C4orf46 /// TOMM7 | chromosome 4 open reading frame 46 /// translocase of outer mitochondrial membrane 7 homolog (yeast) |
| C5orf54 | chromosome 5 open reading frame 54 |
| C6orf57 | chromosome 6 open reading frame 57 |
| C7orf55 | chromosome 7 open reading frame 55 |
| C7orf55 /// LUC7L2 | chromosome 7 open reading frame 55 /// LUC7-like 2 (S. cerevisiae) |
| C9orf46 | chromosome 9 open reading frame 46 |
| CA5B | carbonic anhydrase VB, mitochondrial |
| CA5BP | Carbonic anhydrase VB pseudogene |
| CAV1 | caveolin 1, caveolae protein, 22kDa |
| CBARA1 | calcium binding atopy-related autoantigen 1 |
| CCBL2 | cysteine conjugate-beta lyase 2 |
| CCDC123 | coiled-coil domain containing 123 |
| CCDC58 | coiled-coil domain containing 58 |
| CCT7 | chaperonin containing TCP1, subunit 7 (eta) |
| CDS2 /// LOC149832 | CDP-diacylglycerol synthase (phosphatidate cytidylyltransferase) 2 |
| CECR5 | cat eye syndrome chromosome region, candidate 5 |
| CHCHD1 | coiled-coil-helix-coiled-coil-helix domain containing 1 |
| CHCHD2 | coiled-coil-helix-coiled-coil-helix domain containing 2 |
| CHCHD7 | coiled-coil-helix-coiled-coil-helix domain containing 7 |
| CISD2 | CDGSH iron sulfur domain 2 |
| CISD3 | CDGSH iron sulfur domain 3 |
| CKMT1A /// CKMT1B | creatine kinase, mitochondrial 1A /// creatine kinase, mitochondrial 1B |
| CLN3 | ceroid-lipofuscinosis, neuronal 3 |
| COL4A3BP | collagen, type IV, alpha 3 (Goodpasture antigen) binding protein |
| COMTD1 | catechol-O-methyltransferase domain containing 1 |
| COQ10A | coenzyme Q10 homolog A (S. cerevisiae) |
| COQ3 | coenzyme Q3 homolog, methyltransferase (S. cerevisiae) |
| COQ5 | coenzyme Q5 homolog, methyltransferase (S. cerevisiae) |
| COQ6 | coenzyme Q6 homolog, monooxygenase (S. cerevisiae) |
| COQ9 | coenzyme Q9 homolog (S. cerevisiae) |
| COX10 | COX10 homolog, cytochrome c oxidase assembly protein, heme A: farnesyltransferase (yeast) |
| COX15 | COX15 homolog, cytochrome c oxidase assembly protein (yeast) |
| COX4I1 | cytochrome c oxidase subunit IV isoform 1 |
| COX4NB | COX4 neighbor |
| COX5B | cytochrome c oxidase subunit Vb |
| COX6B1 | cytochrome c oxidase subunit VIb polypeptide 1 (ubiquitous) |
| COX6C | cytochrome c oxidase subunit VIc |
| COX7A2L | cytochrome c oxidase subunit VIIa polypeptide 2 like |
| COX7B | cytochrome c oxidase subunit VIIb |
| COX7C | cytochrome c oxidase subunit VIIc |
| CPOX | coproporphyrinogen oxidase |
| CPS1 | carbamoyl-phosphate synthase 1, mitochondrial |
| CPT2 | carnitine palmitoyltransferase 2 |
| CRAT | carnitine O-acetyltransferase |
| CS | citrate synthase |
| CYB5B | cytochrome b5 type B (outer mitochondrial membrane) |
| CYC1 | cytochrome c-1 |
| CYP1A1 | cytochrome P450, family 1, subfamily A, polypeptide 1 |
| CYP24A1 | cytochrome P450, family 24, subfamily A, polypeptide 1 |
| DAB1 /// OMA1 | disabled homolog 1 (Drosophila) /// OMA1 homolog, zinc metallopeptidase (S. cerevisiae) |
| DARS2 | aspartyl-tRNA synthetase 2, mitochondrial |
| DBI | diazepam binding inhibitor (GABA receptor modulator, acyl-CoA binding protein) |
| DECR1 | 2,4-dienoyl CoA reductase 1, mitochondrial |
| DHODH | dihydroorotate dehydrogenase |
| DHRS4 /// DHRS4L2 | dehydrogenase/reductase (SDR family) member 4 /// dehydrogenase/reductase (SDR family) member 4 like 2 |
| DLD | dihydrolipoamide dehydrogenase |
| DLST | dihydrolipoamide S-succinyltransferase (E2 component of 2-oxo-glutarate complex) |
| DNAJA3 | DnaJ (Hsp40) homolog, subfamily A, member 3 |
| DNAJC19 | DnaJ (Hsp40) homolog, subfamily C, member 19 |
| DNLZ | DNL-type zinc finger |
| DNM3 | dynamin 3 |
| DRG2 | developmentally regulated GTP binding protein 2 |
| DYNLL1 | dynein, light chain, LC8-type 1 |
| EARS2 | glutamyl-tRNA synthetase 2, mitochondrial (putative) |
| ECH1 | enoyl CoA hydratase 1, peroxisomal |
| EHHADH | enoyl-CoA, hydratase/3-hydroxyacyl CoA dehydrogenase |
| ELAC2 | elaC homolog 2 (E. coli) |
| ENDOG | endonuclease G |
| ENO1 | enolase 1, (alpha) |
| ETFA | electron-transfer-flavoprotein, alpha polypeptide |
| ETFB | electron-transfer-flavoprotein, beta polypeptide |
| FAHD1 | fumarylacetoacetate hydrolase domain containing 1 |
| FAM110B | family with sequence similarity 110, member B |
| FAM136A | family with sequence similarity 136, member A |
| FAM175A | family with sequence similarity 175, member A |
| FAM82B | Family with sequence similarity 82, member B |
| FANCG | Fanconi anemia, complementation group G |
| FARS2 | Phenylalanyl-tRNA synthetase 2, mitochondrial |
| FASTK | Fas-activated serine/threonine kinase |
| FDPS | farnesyl diphosphate synthase (farnesyl pyrophosphate synthetase, dimethylallyltranstransferase, geranyltranstransferase) |
| FDX1 | ferredoxin 1 |
| FECH | ferrochelatase |
| FEZ1 | fasciculation and elongation protein zeta 1 (zygin I) |
| FIBP | fibroblast growth factor (acidic) intracellular binding protein |
| FIS1 | fission 1 (mitochondrial outer membrane) homolog (S. cerevisiae) |
| FUNDC2 | FUN14 domain containing 2 |
| FXC1 | fracture callus 1 homolog (rat) |
| FXN | frataxin |
| GABBR1 /// UBD | gamma-aminobutyric acid (GABA) B receptor, 1 /// ubiquitin D |
| GAD1 | glutamate decarboxylase 1 (brain, 67kDa) |
| GADD45GIP1 | growth arrest and DNA-damage-inducible, gamma interacting protein 1 |
| GAS8 | growth arrest-specific 8 |
| GBAS | glioblastoma amplified sequence |
| GCDH | glutaryl-CoA dehydrogenase |
| GHITM | growth hormone inducible transmembrane protein |
| GJA1 | gap junction protein, alpha 1, 43kDa |
| GK | glycerol kinase |
| GK /// GK3P | glycerol kinase /// glycerol kinase 3 pseudogene |
| GK3P | glycerol kinase 3 pseudogene |
| GLOD4 | glyoxalase domain containing 4 |
| GLS2 | glutaminase 2 (liver, mitochondrial) |
| GLT8D1 | Glycosyltransferase 8 domain containing 1 |
| GLYCTK | glycerate kinase |
| GM2A | GM2 ganglioside activator |
| GNPAT | glyceronephosphate O-acyltransferase |
| GPX4 | glutathione peroxidase 4 (phospholipid hydroperoxidase) |
| GRAMD4 | GRAM domain containing 4 |
| GRN | granulin |
| GTF2H4 | general transcription factor IIH, polypeptide 4, 52kDa |
| GTPBP3 | GTP binding protein 3 (mitochondrial) |
| HCCS | holocytochrome c synthase |
| HDDC2 | HD domain containing 2 |
| HEBP1 | heme binding protein 1 |
| HEBP2 | heme binding protein 2 |
| HEMK1 | HemK methyltransferase family member 1 |
| HIBADH | 3-hydroxyisobutyrate dehydrogenase |
| HINT2 | histidine triad nucleotide binding protein 2 |
| HK1 | hexokinase 1 |
| HK2 | hexokinase 2 |
| HLCS | holocarboxylase synthetase (biotin-(proprionyl-CoA-carboxylase (ATP-hydrolysing)) ligase) |
| HRSP12 | Heat-responsive protein 12 |
| HSD17B10 | hydroxysteroid (17-beta) dehydrogenase 10 |
| HSD17B8 | hydroxysteroid (17-beta) dehydrogenase 8 |
| HSP90AB1 | heat shock protein 90kDa alpha (cytosolic), class B member 1 |
| HSPE1 | heat shock 10kDa protein 1 (chaperonin 10) |
| ICT1 | immature colon carcinoma transcript 1 |
| IDH1 | Isocitrate dehydrogenase 1 (NADP+), soluble |
| IDH2 | isocitrate dehydrogenase 2 (NADP+), mitochondrial |
| IDH3A | isocitrate dehydrogenase 3 (NAD+) alpha |
| IDH3B | isocitrate dehydrogenase 3 (NAD+) beta |
| IMMP2L | IMP2 inner mitochondrial membrane peptidase-like (S. cerevisiae) |
| ISCA1 | iron-sulfur cluster assembly 1 homolog (S. cerevisiae) |
| ISOC2 | isochorismatase domain containing 2 |
| KARS | lysyl-tRNA synthetase |
| KIAA0141 | KIAA0141 |
| KMO | kynurenine 3-monooxygenase (kynurenine 3-hydroxylase) |
| KRT5 | keratin 5 |
| L2HGDH | L-2-hydroxyglutarate dehydrogenase |
| LARS2 | leucyl-tRNA synthetase 2, mitochondrial |
| LETM1 | leucine zipper-EF-hand containing transmembrane protein 1 |
| LETMD1 | LETM1 domain containing 1 |
| LGALS3 | Lectin, galactoside-binding, soluble, 3 |
| LIMK2 | LIM domain kinase 2 |
| LIPT1 | lipoyltransferase 1 |
| LONP1 | lon peptidase 1, mitochondrial |
| LRP5 | Low density lipoprotein receptor-related protein 5 |
| LYRM1 | LYR motif containing 1 |
| LYRM2 | LYR motif containing 2 |
| LYRM4 | LYR motif containing 4 |
| LYRM5 | LYR motif containing 5 |
| MARS2 | methionyl-tRNA synthetase 2, mitochondrial |
| MAT2B | Methionine adenosyltransferase II, beta |
| MAVS | mitochondrial antiviral signaling protein |
| MCAT | malonyl CoA:ACP acyltransferase (mitochondrial) |
| MCEE | methylmalonyl CoA epimerase |
| MCL1 | myeloid cell leukemia sequence 1 (BCL2-related) |
| ME3 | malic enzyme 3, NADP(+)-dependent, mitochondrial |
| MECR | mitochondrial trans-2-enoyl-CoA reductase |
| MFF | mitochondrial fission factor |
| MIPEP | mitochondrial intermediate peptidase |
| MLXIP | MLX interacting protein |
| MMACHC | methylmalonic aciduria (cobalamin deficiency) cblC type, with homocystinuria |
| MOSC1 | MOCO sulphurase C-terminal domain containing 1 |
| MPST | mercaptopyruvate sulfurtransferase |
| MPV17 | MpV17 mitochondrial inner membrane protein |
| MPV17L2 | MPV17 mitochondrial membrane protein-like 2 |
| MRPL1 | mitochondrial ribosomal protein L1 |
| MRPL11 | mitochondrial ribosomal protein L11 |
| MRPL12 | Mitochondrial ribosomal protein L12 |
| MRPL13 | mitochondrial ribosomal protein L13 |
| MRPL16 | mitochondrial ribosomal protein L16 |
| MRPL18 | mitochondrial ribosomal protein L18 |
| MRPL19 | mitochondrial ribosomal protein L19 |
| MRPL2 | mitochondrial ribosomal protein L2 |
| MRPL20 | mitochondrial ribosomal protein L20 |
| MRPL21 | mitochondrial ribosomal protein L21 |
| MRPL22 | mitochondrial ribosomal protein L22 |
| MRPL23 | mitochondrial ribosomal protein L23 |
| MRPL30 | mitochondrial ribosomal protein L30 |
| MRPL32 | mitochondrial ribosomal protein L32 |
| MRPL35 | mitochondrial ribosomal protein L35 |
| MRPL4 | mitochondrial ribosomal protein L4 |
| MRPL40 | mitochondrial ribosomal protein L40 |
| MRPL41 | mitochondrial ribosomal protein L41 |
| MRPL42 | mitochondrial ribosomal protein L42 |
| MRPL45 | mitochondrial ribosomal protein L45 |
| MRPL48 | mitochondrial ribosomal protein L48 |
| MRPL49 | mitochondrial ribosomal protein L49 |
| MRPL50 | mitochondrial ribosomal protein L50 |
| MRPL51 /// SPTLC1 | mitochondrial ribosomal protein L51 /// serine palmitoyltransferase, long chain base subunit 1 |
| MRPL55 | mitochondrial ribosomal protein L55 |
| MRPL9 | mitochondrial ribosomal protein L9 |
| MRPS12 | mitochondrial ribosomal protein S12 |
| MRPS14 | mitochondrial ribosomal protein S14 |
| MRPS17 /// ZNF713 | mitochondrial ribosomal protein S17 /// zinc finger protein 713 |
| MRPS18A | mitochondrial ribosomal protein S18A |
| MRPS2 | mitochondrial ribosomal protein S2 |
| MRPS21 | mitochondrial ribosomal protein S21 |
| MRPS23 | mitochondrial ribosomal protein S23 |
| MRPS25 | mitochondrial ribosomal protein S25 |
| MRPS27 | mitochondrial ribosomal protein S27 |
| MRPS28 | mitochondrial ribosomal protein S28 |
| MRPS31 | mitochondrial ribosomal protein S31 |
| MRPS33 | mitochondrial ribosomal protein S33 |
| MRPS34 | mitochondrial ribosomal protein S34 |
| MRPS7 | mitochondrial ribosomal protein S7 |
| MRPS9 | mitochondrial ribosomal protein S9 |
| MSRB2 | methionine sulfoxide reductase B2 |
| MSTO1 | misato homolog 1 (Drosophila) |
| MTERFD1 | MTERF domain containing 1 |
| MTERFD3 | MTERF domain containing 3 |
| MTG1 | mitochondrial GTPase 1 homolog (S. cerevisiae) |
| MTHFD1L | methylenetetrahydrofolate dehydrogenase (NADP+ dependent) 1-like |
| MTIF2 | mitochondrial translational initiation factor 2 |
| MTOR | mechanistic target of rapamycin (serine/threonine kinase) |
| MTUS1 | Microtubule associated tumor suppressor 1 |
| MUT | methylmalonyl CoA mutase |
| MUTYH | mutY homolog (E. coli) |
| NCRNA00219 | non-protein coding RNA 219 |
| NDUFA1 | NADH dehydrogenase (ubiquinone) 1 alpha subcomplex, 1, 7.5kDa |
| NDUFA10 | NADH dehydrogenase (ubiquinone) 1 alpha subcomplex, 10, 42kDa |
| NDUFA11 | NADH dehydrogenase (ubiquinone) 1 alpha subcomplex, 11, 14.7kDa |
| NDUFA12 | NADH dehydrogenase (ubiquinone) 1 alpha subcomplex, 12 |
| NDUFA13 | NADH dehydrogenase (ubiquinone) 1 alpha subcomplex, 13 |
| NDUFA2 | NADH dehydrogenase (ubiquinone) 1 alpha subcomplex, 2, 8kDa |
| NDUFA3 | NADH dehydrogenase (ubiquinone) 1 alpha subcomplex, 3, 9kDa |
| NDUFA4 | NADH dehydrogenase (ubiquinone) 1 alpha subcomplex, 4, 9kDa |
| NDUFA6 | NADH dehydrogenase (ubiquinone) 1 alpha subcomplex, 6, 14kDa |
| NDUFA7 | NADH dehydrogenase (ubiquinone) 1 alpha subcomplex, 7, 14.5kDa |
| NDUFA8 | NADH dehydrogenase (ubiquinone) 1 alpha subcomplex, 8, 19kDa |
| NDUFA9 | NADH dehydrogenase (ubiquinone) 1 alpha subcomplex, 9, 39kDa |
| NDUFAB1 | NADH dehydrogenase (ubiquinone) 1, alpha/beta subcomplex, 1, 8kDa |
| NDUFAF2 | NADH dehydrogenase (ubiquinone) 1 alpha subcomplex, assembly factor 2 |
| NDUFB3 | NADH dehydrogenase (ubiquinone) 1 beta subcomplex, 3, 12kDa |
| NDUFB5 | NADH dehydrogenase (ubiquinone) 1 beta subcomplex, 5, 16kDa |
| NDUFB7 | NADH dehydrogenase (ubiquinone) 1 beta subcomplex, 7, 18kDa |
| NDUFB8 | NADH dehydrogenase (ubiquinone) 1 beta subcomplex, 8, 19kDa |
| NDUFB9 | NADH dehydrogenase (ubiquinone) 1 beta subcomplex, 9, 22kDa |
| NDUFC2 | NADH dehydrogenase (ubiquinone) 1, subcomplex unknown, 2, 14.5kDa |
| NDUFS5 /// RPL10 | NADH dehydrogenase (ubiquinone) Fe-S protein 5, (NADH-coenzyme Q reductase) /// ribosomal protein L10 |
| NDUFS7 | NADH dehydrogenase (ubiquinone) Fe-S protein 7, 20kDa (NADH-coenzyme Q reductase) |
| NDUFV1 | NADH dehydrogenase (ubiquinone) flavoprotein 1, 51kDa |
| NDUFV3 | NADH dehydrogenase (ubiquinone) flavoprotein 3, 10kDa |
| NEFH | neurofilament, heavy polypeptide |
| NFS1 | NFS1 nitrogen fixation 1 homolog (S. cerevisiae) |
| NFU1 | NFU1 iron-sulfur cluster scaffold homolog (S. cerevisiae) |
| NIT2 | nitrilase family, member 2 |
| NLRX1 | NLR family member X1 |
| NOP14 | NOP14 nucleolar protein homolog (yeast) |
| NR3C1 | nuclear receptor subfamily 3, group C, member 1 (glucocorticoid receptor) |
| NRAS | neuroblastoma RAS viral (v-ras) oncogene homolog |
| NRD1 | nardilysin (N-arginine dibasic convertase) |
| NT5DC3 | 5'-nucleotidase domain containing 3 |
| NUDT13 | nudix (nucleoside diphosphate linked moiety X)-type motif 13 |
| NUDT19 | nudix (nucleoside diphosphate linked moiety X)-type motif 19 |
| NUDT6 | nudix (nucleoside diphosphate linked moiety X)-type motif 6 |
| NUDT9 | nudix (nucleoside diphosphate linked moiety X)-type motif 9 |
| OAT | ornithine aminotransferase |
| OCIAD1 | OCIA domain containing 1 |
| OGDH | oxoglutarate (alpha-ketoglutarate) dehydrogenase (lipoamide) |
| OGG1 | 8-oxoguanine DNA glycosylase |
| OMA1 | OMA1 homolog, zinc metallopeptidase (S. cerevisiae) |
| OXA1L | oxidase (cytochrome c) assembly 1-like |
| OXR1 | oxidation resistance 1 |
| OXSM | 3-oxoacyl-ACP synthase, mitochondrial |
| P4HA1 | prolyl 4-hydroxylase, alpha polypeptide I |
| PACS2 | phosphofurin acidic cluster sorting protein 2 |
| PARG | poly (ADP-ribose) glycohydrolase |
| PARK7 | Parkinson disease (autosomal recessive, early onset) 7 |
| PARL | presenilin associated, rhomboid-like |
| PARS2 | prolyl-tRNA synthetase 2, mitochondrial (putative) |
| PCCB | propionyl CoA carboxylase, beta polypeptide |
| PCK2 | phosphoenolpyruvate carboxykinase 2 (mitochondrial) |
| PDK1 | pyruvate dehydrogenase kinase, isozyme 1 |
| PDSS2 | prenyl (decaprenyl) diphosphate synthase, subunit 2 |
| PECI | peroxisomal D3,D2-enoyl-CoA isomerase |
| PEMT | phosphatidylethanolamine N-methyltransferase |
| PERP | PERP, TP53 apoptosis effector |
| PET112L | PET112-like (yeast) |
| PGAM5 | phosphoglycerate mutase family member 5 |
| PHYH | phytanoyl-CoA 2-hydroxylase |
| PHYHIPL | phytanoyl-CoA 2-hydroxylase interacting protein-like |
| PICK1 | protein interacting with PRKCA 1 |
| PIGY | phosphatidylinositol glycan anchor biosynthesis, class Y |
| PINK1 | PTEN induced putative kinase 1 |
| PITRM1 | pitrilysin metallopeptidase 1 |
| PMPCA | peptidase (mitochondrial processing) alpha |
| PNKD | paroxysmal nonkinesigenic dyskinesia |
| POLDIP2 | polymerase (DNA-directed), delta interacting protein 2 |
| POLG | polymerase (DNA directed), gamma |
| POLG2 | polymerase (DNA directed), gamma 2, accessory subunit |
| POLRMT | polymerase (RNA) mitochondrial (DNA directed) |
| PON2 | paraoxonase 2 |
| PPIF | peptidylprolyl isomerase F |
| PPOX | protoporphyrinogen oxidase |
| PPP1CA | protein phosphatase 1, catalytic subunit, alpha isozyme |
| PPP2CA | protein phosphatase 2, catalytic subunit, alpha isozyme |
| PPP3CB | protein phosphatase 3, catalytic subunit, beta isozyme |
| PRDX1 | peroxiredoxin 1 |
| PRDX2 | peroxiredoxin 2 |
| PRDX5 | peroxiredoxin 5 |
| PRKACA | protein kinase, cAMP-dependent, catalytic, alpha |
| PTGR2 | prostaglandin reductase 2 |
| PTRF | polymerase I and transcript release factor |
| PTRH1 | Peptidyl-tRNA hydrolase 1 homolog (S. cerevisiae) |
| PTRH2 | peptidyl-tRNA hydrolase 2 |
| PTS | 6-pyruvoyltetrahydropterin synthase |
| PUS1 | pseudouridylate synthase 1 |
| PYCR1 | pyrroline-5-carboxylate reductase 1 |
| QTRT1 | queuine tRNA-ribosyltransferase 1 |
| QTRTD1 | queuine tRNA-ribosyltransferase domain containing 1 |
| RAF1 | v-raf-1 murine leukemia viral oncogene homolog 1 |
| RAI14 | retinoic acid induced 14 |
| RARS2 | arginyl-tRNA synthetase 2, mitochondrial |
| RBFA | ribosome binding factor A |
| RDBP | RD RNA binding protein |
| RDH13 | Retinol dehydrogenase 13 (all-trans/9-cis) |
| RG9MTD1 | RNA (guanine-9-) methyltransferase domain containing 1 |
| RNF5 | ring finger protein 5 |
| RPL9 | ribosomal protein L9 |
| RPP21 /// TRIM39 /// TRIM39R | ribonuclease P/MRP 21kDa subunit /// tripartite motif-containing 39 /// TRIM39-like protein |
| RPS6KB1 | ribosomal protein S6 kinase, 70kDa, polypeptide 1 |
| RPUSD4 | RNA pseudouridylate synthase domain containing 4 |
| SAMM50 | sorting and assembly machinery component 50 homolog (S. cerevisiae) |
| SARS | Seryl-tRNA synthetase |
| SCCPDH | saccharopine dehydrogenase (putative) |
| SDHA | succinate dehydrogenase complex, subunit A, flavoprotein (Fp) |
| SDHA /// SDHAP1 /// SDHAP2 | succinate dehydrogenase complex, subunit A, flavoprotein (Fp) /// succinate dehydrogenase complex, subunit A, flavoprotein pseudogene 1 /// succinate dehydrogenase complex, subunit A, flavoprotein pseudogene 2 |
| SDHAF1 | succinate dehydrogenase complex assembly factor 1 |
| SDHC | succinate dehydrogenase complex, subunit C, integral membrane protein, 15kDa |
| SDHD | succinate dehydrogenase complex, subunit D, integral membrane protein |
| SFXN1 | sideroflexin 1 |
| SFXN2 | sideroflexin 2 |
| SHC1 | SHC (Src homology 2 domain containing) transforming protein 1 |
| SIRT5 | sirtuin (silent mating type information regulation 2 homolog) 5 (S. cerevisiae) |
| SLC1A3 | solute carrier family 1 (glial high affinity glutamate transporter), member 3 |
| SLC25A12 | solute carrier family 25 (mitochondrial carrier, Aralar), member 12 |
| SLC25A14 | solute carrier family 25 (mitochondrial carrier, brain), member 14 |
| SLC25A25 | solute carrier family 25, member 25 |
| SLC25A26 | solute carrier family 25, member 26 |
| SLC25A29 | solute carrier family 25, member 29 |
| SLC25A3 | solute carrier family 25, member 3 |
| SLC25A32 | solute carrier family 25, member 32 |
| SLC25A36 | Solute carrier family 25, member 36 |
| SLC25A38 | solute carrier family 25, member 38 |
| SLC25A39 | solute carrier family 25, member 39 |
| SLC25A44 | solute carrier family 25, member 44 |
| SLC25A6 | solute carrier family 25, member 6 |
| SLC27A3 | solute carrier family 27 (fatty acid transporter), member 3 |
| SLIT3 | slit homolog 3 (Drosophila) |
| SLMO2 | slowmo homolog 2 (Drosophila) |
| SNN | stannin |
| SOD1 | superoxide dismutase 1, soluble |
| STAR | steroidogenic acute regulatory protein |
| STOML2 | stomatin (EPB72)-like 2 |
| SUCLG1 | succinate-CoA ligase, alpha subunit |
| SURF1 | surfeit 1 |
| TATDN3 | TatD DNase domain containing 3 |
| TBC1D15 | TBC1 domain family, member 15 |
| TBRG4 | transforming growth factor beta regulator 4 |
| THG1L | tRNA-histidine guanylyltransferase 1-like (S. cerevisiae) |
| TIMM13 | translocase of inner mitochondrial membrane 13 homolog (yeast) |
| TIMM17A | translocase of inner mitochondrial membrane 17 homolog A (yeast) |
| TIMM22 | Translocase of inner mitochondrial membrane 22 homolog (yeast) |
| TIMM23 /// TIMM23B | translocase of inner mitochondrial membrane 23 homolog (yeast) /// translocase of inner mitochondrial membrane 23 homolog B (yeast) |
| TIMM8A | translocase of inner mitochondrial membrane 8 homolog A (yeast) |
| TMEM126A | transmembrane protein 126A |
| TMEM14B /// TMEM14C | transmembrane protein 14B /// transmembrane protein 14C |
| TMEM14C | transmembrane protein 14C |
| TMEM160 | transmembrane protein 160 |
| TMEM223 | transmembrane protein 223 |
| TMTC1 | transmembrane and tetratricopeptide repeat containing 1 |
| TOMM22 | translocase of outer mitochondrial membrane 22 homolog (yeast) |
| TOMM34 | translocase of outer mitochondrial membrane 34 |
| TOMM70A | translocase of outer mitochondrial membrane 70 homolog A (S. cerevisiae) |
| TP53 | tumor protein p53 |
| TRAP1 | TNF receptor-associated protein 1 |
| TRIT1 | tRNA isopentenyltransferase 1 |
| TRNT1 | tRNA nucleotidyl transferase, CCA-adding, 1 |
| TXNDC12 | thioredoxin domain containing 12 (endoplasmic reticulum) |
| TXNRD1 | thioredoxin reductase 1 |
| UQCC | ubiquinol-cytochrome c reductase complex chaperone |
| UQCR10 | ubiquinol-cytochrome c reductase, complex III subunit X |
| UQCR11 | ubiquinol-cytochrome c reductase, complex III subunit XI |
| UQCRB | ubiquinol-cytochrome c reductase binding protein |
| UQCRH | ubiquinol-cytochrome c reductase hinge protein |
| UQCRQ | ubiquinol-cytochrome c reductase, complex III subunit VII, 9.5kDa |
| USP30 | ubiquitin specific peptidase 30 |
| VARS2 | valyl-tRNA synthetase 2, mitochondrial (putative) |
| VHL | von Hippel-Lindau tumor suppressor |
| WARS2 | tryptophanyl tRNA synthetase 2, mitochondrial |
| WASF1 | WAS protein family, member 1 |
| YME1L1 | YME1-like 1 (S. cerevisiae) |
| YWHAE | Tyrosine 3-monooxygenase/tryptophan 5-monooxygenase activation protein, epsilon polypeptide |
| YWHAZ | tyrosine 3-monooxygenase/tryptophan 5-monooxygenase activation protein, zeta polypeptide |

**Table S5. Genes induced in the lower airways of healthy controls, assigned to GO cluster *Mitochondrion*.**

| **Gene alias** | **Gene name/description** |
| --- | --- |
|  |  |
| ABCB6 | ATP-binding cassette, sub-family B (MDR/TAP), member 6 |
| ACADVL | acyl-CoA dehydrogenase, very long chain |
| ACP6 | acid phosphatase 6, lysophosphatidic |
| ADH5 | alcohol dehydrogenase 5 (class III), chi polypeptide |
| ADO | 2-aminoethanethiol (cysteamine) dioxygenase |
| AIFM1 | apoptosis-inducing factor, mitochondrion-associated, 1 |
| AK1 | adenylate kinase 1 |
| AKT2 | v-akt murine thymoma viral oncogene homolog 2 |
| ALDH18A1 | aldehyde dehydrogenase 18 family, member A1 |
| ALKBH7 | AlkB, alkylation repair homolog 7 (E. coli) |
| ARAF | v-raf murine sarcoma 3611 viral oncogene homolog |
| ARSB | arylsulfatase B |
| ASS1 | argininosuccinate synthase 1 |
| ATP5C1 | ATP synthase, H+ transporting, mitochondrial F1 complex, gamma polypeptide 1 |
| ATP5G3 | ATP synthase, H+ transporting, mitochondrial F0 complex, subunit C3 (subunit 9) |
| ATP5H | ATP synthase, H+ transporting, mitochondrial F0 complex, subunit d |
| ATP5I | ATP synthase, H+ transporting, mitochondrial F0 complex, subunit E |
| ATP5J2 | ATP synthase, H+ transporting, mitochondrial F0 complex, subunit F2 |
| ATP5SL | ATP5S-like |
| ATP6V1A | ATPase, H+ transporting, lysosomal 70kDa, V1 subunit A |
| AURKAIP1 | aurora kinase A interacting protein 1 |
| BCL2L10 | BCL2-like 10 (apoptosis facilitator) |
| BCL2L2 | BCL2-like 2 |
| BLOC1S1 | biogenesis of lysosomal organelles complex-1, subunit 1 |
| BNIP3L | BCL2/adenovirus E1B 19kDa interacting protein 3-like |
| C10orf58 | chromosome 10 open reading frame 58 |
| C12orf10 | chromosome 12 open reading frame 10 |
| C12orf62 | chromosome 12 open reading frame 62 |
| C14orf159 | chromosome 14 open reading frame 159 |
| C15orf62 | chromosome 15 open reading frame 62 |
| C17orf61 | chromosome 17 open reading frame 61 |
| C18orf19 | chromosome 18 open reading frame 19 |
| C1orf31 | chromosome 1 open reading frame 31 |
| C22orf32 | chromosome 22 open reading frame 32 |
| C2orf56 | chromosome 2 open reading frame 56 |
| C9orf46 | chromosome 9 open reading frame 46 |
| CA5B | carbonic anhydrase VB, mitochondrial |
| CCDC142 /// MRPL53 | coiled-coil domain containing 142 /// mitochondrial ribosomal protein L53 |
| CCDC58 | coiled-coil domain containing 58 |
| CDS2 /// LOC149832 | CDP-diacylglycerol synthase (phosphatidate cytidylyltransferase) 2 |
| CHAF1B | chromatin assembly factor 1, subunit B (p60) |
| CHCHD1 | coiled-coil-helix-coiled-coil-helix domain containing 1 |
| CHCHD2 | coiled-coil-helix-coiled-coil-helix domain containing 2 |
| CHCHD4 | coiled-coil-helix-coiled-coil-helix domain containing 4 |
| CIDEA | cell death-inducing DFFA-like effector a |
| CISD3 | CDGSH iron sulfur domain 3 |
| CLPX | ClpX caseinolytic peptidase X homolog (E. coli) |
| CLTC | Clathrin, heavy chain (Hc) |
| COL4A3BP | collagen, type IV, alpha 3 (Goodpasture antigen) binding protein |
| COMTD1 | catechol-O-methyltransferase domain containing 1 |
| COQ5 | coenzyme Q5 homolog, methyltransferase (S. cerevisiae) |
| COQ9 | coenzyme Q9 homolog (S. cerevisiae) |
| COX10 | COX10 homolog, cytochrome c oxidase assembly protein, heme A: farnesyltransferase (yeast) |
| COX15 | COX15 homolog, cytochrome c oxidase assembly protein (yeast) |
| COX16 | COX16 cytochrome c oxidase assembly homolog (S. cerevisiae) |
| COX4NB | COX4 neighbor |
| COX5B | Cytochrome c oxidase subunit Vb |
| COX6B1 | cytochrome c oxidase subunit VIb polypeptide 1 (ubiquitous) |
| COX7C | cytochrome c oxidase subunit VIIc |
| CRAT | carnitine O-acetyltransferase |
| CROT | carnitine O-octanoyltransferase |
| CXorf23 | chromosome X open reading frame 23 |
| CYB5R1 | cytochrome b5 reductase 1 |
| CYB5R3 | cytochrome b5 reductase 3 |
| DDX28 | DEAD (Asp-Glu-Ala-Asp) box polypeptide 28 |
| DHODH | dihydroorotate dehydrogenase |
| DHX29 | DEAH (Asp-Glu-Ala-His) box polypeptide 29 |
| DHX30 | DEAH (Asp-Glu-Ala-His) box polypeptide 30 |
| DLST | dihydrolipoamide S-succinyltransferase (E2 component of 2-oxo-glutarate complex) |
| DNAJC15 | DnaJ (Hsp40) homolog, subfamily C, member 15 |
| DNAJC4 | DnaJ (Hsp40) homolog, subfamily C, member 4 |
| DPYSL2 | dihydropyrimidinase-like 2 |
| DRG2 | developmentally regulated GTP binding protein 2 |
| DYNLL1 | dynein, light chain, LC8-type 1 |
| EHHADH | enoyl-CoA, hydratase/3-hydroxyacyl CoA dehydrogenase |
| ELAC2 | elaC homolog 2 (E. coli) |
| ENDOG | endonuclease G |
| ENO1 | enolase 1, (alpha) |
| FAHD1 | fumarylacetoacetate hydrolase domain containing 1 |
| FASTK | Fas-activated serine/threonine kinase |
| FOXRED1 | FAD-dependent oxidoreductase domain containing 1 |
| FTSJ2 | FtsJ homolog 2 (E. coli) |
| FUNDC2 | FUN14 domain containing 2 |
| FXC1 | fracture callus 1 homolog (rat) |
| FXN | frataxin |
| GADD45GIP1 | Growth arrest and DNA-damage-inducible, gamma interacting protein 1 |
| GATC | Glutamyl-tRNA(Gln) amidotransferase, subunit C homolog (bacterial) |
| GATM | Glycine amidinotransferase (L-arginine:glycine amidinotransferase) |
| GHITM | growth hormone inducible transmembrane protein |
| GK /// GK3P | glycerol kinase /// glycerol kinase 3 pseudogene |
| GK3P | glycerol kinase 3 pseudogene |
| GLUL | glutamate-ammonia ligase |
| GLYCTK | glycerate kinase |
| GNPAT | glyceronephosphate O-acyltransferase |
| GOT2 | glutamic-oxaloacetic transaminase 2, mitochondrial (aspartate aminotransferase 2) |
| GRN | granulin |
| GRPEL1 | GrpE-like 1, mitochondrial (E. coli) |
| GRPEL2 | GrpE-like 2, mitochondrial (E. coli) |
| GSTZ1 | glutathione transferase zeta 1 |
| GTF2H4 | general transcription factor IIH, polypeptide 4, 52kDa |
| GTPBP5 | GTP binding protein 5 (putative) |
| GTPBP8 | GTP-binding protein 8 (putative) |
| HEBP2 | heme binding protein 2 |
| HEMK1 | HemK methyltransferase family member 1 |
| HK2 | hexokinase 2 |
| HSCB | HscB iron-sulfur cluster co-chaperone homolog (E. coli) |
| HTRA2 | HtrA serine peptidase 2 |
| ICT1 | immature colon carcinoma transcript 1 |
| IDH1 | Isocitrate dehydrogenase 1 (NADP+), soluble |
| IDH3B | isocitrate dehydrogenase 3 (NAD+) beta |
| ISCA1 | iron-sulfur cluster assembly 1 homolog (S. cerevisiae) |
| ISCA2 | iron-sulfur cluster assembly 2 homolog (S. cerevisiae) |
| IVD | isovaleryl-CoA dehydrogenase |
| JMJD7 | jumonji domain containing 7 |
| KIAA0141 | KIAA0141 |
| KIF1B | kinesin family member 1B |
| KMO | kynurenine 3-monooxygenase (kynurenine 3-hydroxylase) |
| LARS2 | leucyl-tRNA synthetase 2, mitochondrial |
| LETM1 | leucine zipper-EF-hand containing transmembrane protein 1 |
| LETMD1 | LETM1 domain containing 1 |
| LGALS3 | lectin, galactoside-binding, soluble, 3 |
| LIMK2 | LIM domain kinase 2 |
| LOC100510009 /// WBSCR16 | Williams-Beuren syndrome chromosome region 16 |
| LYRM2 | LYR motif containing 2 |
| MAVS | mitochondrial antiviral signaling protein |
| MCAT | malonyl CoA:ACP acyltransferase (mitochondrial) |
| MCL1 | myeloid cell leukemia sequence 1 (BCL2-related) |
| MECR | mitochondrial trans-2-enoyl-CoA reductase |
| MMACHC | methylmalonic aciduria (cobalamin deficiency) cblC type, with homocystinuria |
| MRPL11 | mitochondrial ribosomal protein L11 |
| MRPL13 | mitochondrial ribosomal protein L13 |
| MRPL16 | mitochondrial ribosomal protein L16 |
| MRPL17 | mitochondrial ribosomal protein L17 |
| MRPL18 | mitochondrial ribosomal protein L18 |
| MRPL20 | mitochondrial ribosomal protein L20 |
| MRPL22 | mitochondrial ribosomal protein L22 |
| MRPL32 | mitochondrial ribosomal protein L32 |
| MRPL33 | mitochondrial ribosomal protein L33 |
| MRPL36 | mitochondrial ribosomal protein L36 |
| MRPL4 | mitochondrial ribosomal protein L4 |
| MRPL41 | mitochondrial ribosomal protein L41 |
| MRPL43 | mitochondrial ribosomal protein L43 |
| MRPL45 | mitochondrial ribosomal protein L45 |
| MRPL50 | mitochondrial ribosomal protein L50 |
| MRPL55 | Mitochondrial ribosomal protein L55 |
| MRPL9 | mitochondrial ribosomal protein L9 |
| MRPS12 | Mitochondrial ribosomal protein S12 |
| MRPS14 | mitochondrial ribosomal protein S14 |
| MRPS15 | mitochondrial ribosomal protein S15 |
| MRPS17 /// ZNF713 | mitochondrial ribosomal protein S17 /// zinc finger protein 713 |
| MRPS2 | mitochondrial ribosomal protein S2 |
| MRPS24 | mitochondrial ribosomal protein S24 |
| MRPS26 | mitochondrial ribosomal protein S26 |
| MRPS28 | mitochondrial ribosomal protein S28 |
| MRPS9 | mitochondrial ribosomal protein S9 |
| MSTO1 /// MSTO2P | misato homolog 1 (Drosophila) /// misato homolog 2 pseudogene |
| MTFMT | mitochondrial methionyl-tRNA formyltransferase |
| MTG1 | mitochondrial GTPase 1 homolog (S. cerevisiae) |
| MTOR | Mechanistic target of rapamycin (serine/threonine kinase) |
| MUT | methylmalonyl CoA mutase |
| NAGS | N-acetylglutamate synthase |
| NDUFA1 | NADH dehydrogenase (ubiquinone) 1 alpha subcomplex, 1, 7.5kDa |
| NDUFA11 | NADH dehydrogenase (ubiquinone) 1 alpha subcomplex, 11, 14.7kDa |
| NDUFA12 | NADH dehydrogenase (ubiquinone) 1 alpha subcomplex, 12 |
| NDUFA13 | NADH dehydrogenase (ubiquinone) 1 alpha subcomplex, 13 |
| NDUFA3 | NADH dehydrogenase (ubiquinone) 1 alpha subcomplex, 3, 9kDa |
| NDUFA4 | NADH dehydrogenase (ubiquinone) 1 alpha subcomplex, 4, 9kDa |
| NDUFA8 | NADH dehydrogenase (ubiquinone) 1 alpha subcomplex, 8, 19kDa |
| NDUFAB1 | NADH dehydrogenase (ubiquinone) 1, alpha/beta subcomplex, 1, 8kDa |
| NDUFB1 | NADH dehydrogenase (ubiquinone) 1 beta subcomplex, 1, 7kDa |
| NDUFB3 | NADH dehydrogenase (ubiquinone) 1 beta subcomplex, 3, 12kDa |
| NDUFB5 | NADH dehydrogenase (ubiquinone) 1 beta subcomplex, 5, 16kDa |
| NDUFB7 | NADH dehydrogenase (ubiquinone) 1 beta subcomplex, 7, 18kDa |
| NDUFB9 | NADH dehydrogenase (ubiquinone) 1 beta subcomplex, 9, 22kDa |
| NDUFS5 /// RPL10 | NADH dehydrogenase (ubiquinone) Fe-S protein 5, 15kDa (NADH-coenzyme Q reductase) /// ribosomal protein L10 |
| NDUFS6 | NADH dehydrogenase (ubiquinone) Fe-S protein 6, 13kDa (NADH-coenzyme Q reductase) |
| NFS1 | NFS1 nitrogen fixation 1 homolog (S. cerevisiae) |
| NLRX1 | NLR family member X1 |
| NR3C1 | nuclear receptor subfamily 3, group C, member 1 (glucocorticoid receptor) |
| NRAS | neuroblastoma RAS viral (v-ras) oncogene homolog |
| NRD1 | nardilysin (N-arginine dibasic convertase) |
| NT5C | 5', 3'-nucleotidase, cytosolic |
| OAT | ornithine aminotransferase |
| OCIAD1 | OCIA domain containing 1 |
| OXSM | 3-oxoacyl-ACP synthase, mitochondrial |
| P4HA1 | prolyl 4-hydroxylase, alpha polypeptide I |
| PAM16 | presequence translocase-associated motor 16 homolog |
| PANK2 | Pantothenate kinase 2 |
| PARG | poly (ADP-ribose) glycohydrolase |
| PARL | presenilin associated, rhomboid-like |
| PARS2 | prolyl-tRNA synthetase 2, mitochondrial (putative) |
| PCCA | Propionyl Coenzyme A carboxylase, alpha polypeptide |
| PDK1 | pyruvate dehydrogenase kinase, isozyme 1 |
| PDSS2 | prenyl (decaprenyl) diphosphate synthase, subunit 2 |
| PECR | peroxisomal trans-2-enoyl-CoA reductase |
| PET112L | PET112-like (yeast) |
| PIN4 | protein (peptidylprolyl cis/trans isomerase) NIMA-interacting, 4 (parvulin) |
| PMPCA | peptidase (mitochondrial processing) alpha |
| POLDIP2 | polymerase (DNA-directed), delta interacting protein 2 |
| PPP1CA | protein phosphatase 1, catalytic subunit, alpha isozyme |
| PPP1CC | protein phosphatase 1, catalytic subunit, gamma isozyme |
| PPP3CA | protein phosphatase 3, catalytic subunit, alpha isozyme |
| PROSC | Proline synthetase co-transcribed homolog (bacterial) |
| PTRH1 | peptidyl-tRNA hydrolase 1 homolog (S. cerevisiae) |
| PTRH2 | peptidyl-tRNA hydrolase 2 |
| PUS1 | pseudouridylate synthase 1 |
| RAB11A | RAB11A, member RAS oncogene family |
| RAB3D | RAB3D, member RAS oncogene family |
| RAI14 | retinoic acid induced 14 |
| RARS2 | arginyl-tRNA synthetase 2, mitochondrial |
| RBFA | ribosome binding factor A |
| RDBP | RD RNA binding protein |
| RDH13 | Retinol dehydrogenase 13 (all-trans/9-cis) |
| RNASEL | ribonuclease L (2',5'-oligoisoadenylate synthetase-dependent) |
| RNF5 | ring finger protein 5 |
| RPP21 /// TRIM39 /// TRIM39R | ribonuclease P/MRP 21kDa subunit /// tripartite motif-containing 39 /// TRIM39-like protein |
| SACS | spastic ataxia of Charlevoix-Saguenay (sacsin) |
| SARDH | Sarcosine dehydrogenase |
| SARS2 | seryl-tRNA synthetase 2, mitochondrial |
| SEC61A1 | Sec61 alpha 1 subunit (S. cerevisiae) |
| SFXN5 | sideroflexin 5 |
| SH3BP5 | SH3-domain binding protein 5 (BTK-associated) |
| SIVA1 | SIVA1, apoptosis-inducing factor |
| SLC1A3 | solute carrier family 1 (glial high affinity glutamate transporter), member 3 |
| SLC25A13 | Solute carrier family 25, member 13 (citrin) |
| SLC25A14 | solute carrier family 25 (mitochondrial carrier, brain), member 14 |
| SLC25A25 | solute carrier family 25, member 25 |
| SLC25A26 | solute carrier family 25, member 26 |
| SLC25A29 | Solute carrier family 25, member 29 |
| SLC25A30 | solute carrier family 25, member 30 |
| SLC25A32 | solute carrier family 25, member 32 |
| SLC25A38 | solute carrier family 25, member 38 |
| SLC25A44 | solute carrier family 25, member 44 |
| SLC25A45 | solute carrier family 25, member 45 |
| SLC27A1 | solute carrier family 27 (fatty acid transporter), member 1 |
| SLC27A3 | solute carrier family 27 (fatty acid transporter), member 3 |
| SLMO2 | slowmo homolog 2 (Drosophila) |
| SND1 | staphylococcal nuclease and tudor domain containing 1 |
| SOD1 | superoxide dismutase 1, soluble |
| SRGAP2 | SLIT-ROBO Rho GTPase activating protein 2 |
| SSSCA1 | Sjogren syndrome/scleroderma autoantigen 1 |
| STAR | steroidogenic acute regulatory protein |
| SURF1 | surfeit 1 |
| SYNJ2BP | synaptojanin 2 binding protein |
| TAOK3 | TAO kinase 3 |
| TATDN3 | TatD DNase domain containing 3 |
| TBC1D15 | TBC1 domain family, member 15 |
| TBRG4 | transforming growth factor beta regulator 4 |
| TDRKH | tudor and KH domain containing |
| TIMM17A | translocase of inner mitochondrial membrane 17 homolog A (yeast) |
| TIMM23 | translocase of inner mitochondrial membrane 23 homolog (yeast) |
| TMEM126A | transmembrane protein 126A |
| TMEM14B /// TMEM14C | transmembrane protein 14B /// transmembrane protein 14C |
| TMEM14C | transmembrane protein 14C |
| TMEM186 | transmembrane protein 186 |
| TMEM223 | transmembrane protein 223 |
| TOMM40 | translocase of outer mitochondrial membrane 40 homolog (yeast) |
| TXNIP | thioredoxin interacting protein |
| UQCR11 | ubiquinol-cytochrome c reductase, complex III subunit XI |
| UQCRC2 | ubiquinol-cytochrome c reductase core protein II |
| UQCRFS1 | ubiquinol-cytochrome c reductase, Rieske iron-sulfur polypeptide 1 |
| WARS2 | tryptophanyl tRNA synthetase 2, mitochondrial |
| ZFHX3 | Zinc finger homeobox 3 |

**Table S6.** Genes induced in the lower airways of healthy controls and allergic rhinitis patients, assigned to GO cluster *Mitochondrion*.

| **Gene alias** | **Gene name/description** |
| --- | --- |
|  |  |
| AASS | aminoadipate-semialdehyde synthase |
| ADCK2 | aarF domain containing kinase 2 |
| AK3 | adenylate kinase 3 |
| AK4 | adenylate kinase 4 |
| ATP5J | ATP synthase, H+ transporting, mitochondrial F0 complex, subunit F6 |
| ATP5L | ATP synthase, H+ transporting, mitochondrial F0 complex, subunit G |
| BBOX1 | butyrobetaine (gamma), 2-oxoglutarate dioxygenase (gamma-butyrobetaine hydroxylase) 1 |
| BCAT1 | branched chain amino-acid transaminase 1, cytosolic |
| BNIP3 | BCL2/adenovirus E1B 19kDa interacting protein 3 |
| C10orf2 | chromosome 10 open reading frame 2 |
| C14orf156 | chromosome 14 open reading frame 156 |
| C19orf70 | chromosome 19 open reading frame 70 |
| C2orf47 | chromosome 2 open reading frame 47 |
| C3orf1 | chromosome 3 open reading frame 1 |
| C3orf78 | small integral membrane protein 4 |
| C4orf14 | chromosome 4 open reading frame 14 |
| C6orf203 | chromosome 6 open reading frame 203 |
| C7orf55 /// LUC7L2 | chromosome 7 open reading frame 55 /// LUC7-like 2 (S. cerevisiae) |
| CCBL2 | cysteine conjugate-beta lyase 2 |
| CCT7 | chaperonin containing TCP1, subunit 7 (eta) |
| CKB | creatine kinase, brain |
| CKMT1A /// CKMT1B | creatine kinase, mitochondrial 1A /// creatine kinase, mitochondrial 1B |
| COX11 | COX11 cytochrome c oxidase assembly homolog (yeast) |
| COX3 | mitochondrially encoded cytochrome c oxidase III |
| COX7A2L | cytochrome c oxidase subunit VIIa polypeptide 2 like |
| CPS1 | carbamoyl-phosphate synthase 1, mitochondrial |
| CS | citrate synthase |
| CYCS | cytochrome c, somatic |
| DARS2 | aspartyl-tRNA synthetase 2, mitochondrial |
| DHRS1 | dehydrogenase/reductase (SDR family) member 1 |
| DLAT | dihydrolipoamide S-acetyltransferase |
| EFHD1 | EF-hand domain family, member D1 |
| FIS1 | fission 1 (mitochondrial outer membrane) homolog (S. cerevisiae) |
| GCAT | glycine C-acetyltransferase |
| GCDH | glutaryl-CoA dehydrogenase |
| GLRX2 | glutaredoxin 2 |
| HERC2 | hect domain and RLD 2 |
| KARS | lysyl-tRNA synthetase |
| KRT4 | keratin 4 |
| LONP1 | lon peptidase 1, mitochondrial |
| LYRM4 | LYR motif containing 4 |
| MARS2 | methionyl-tRNA synthetase 2, mitochondrial |
| MCCC2 | Methylcrotonoyl-Coenzyme A carboxylase 2 (beta) |
| METT11D1 | methyltransferase like 17 |
| METTL12 | methyltransferase like 12 |
| MMAA | methylmalonic aciduria (cobalamin deficiency) cblA type |
| MPST | mercaptopyruvate sulfurtransferase |
| MRPL12 | mitochondrial ribosomal protein L12 |
| MRPL2 | mitochondrial ribosomal protein L2 |
| MRPL21 | mitochondrial ribosomal protein L21 |
| MRPL35 | mitochondrial ribosomal protein L35 |
| MRPL42 | mitochondrial ribosomal protein L42 |
| MRPS16 | mitochondrial ribosomal protein S16 |
| MRPS23 | mitochondrial ribosomal protein S23 |
| MRPS25 | mitochondrial ribosomal protein S25 |
| MRPS33 | mitochondrial ribosomal protein S33 |
| MRPS34 | mitochondrial ribosomal protein S34 |
| MRPS35 | mitochondrial ribosomal protein S35 |
| MRPS7 | mitochondrial ribosomal protein S7 |
| MSTO1 | misato homolog 1 (Drosophila) |
| MTERFD1 | MTERF domain containing 1 |
| MTHFD2 | methylenetetrahydrofolate dehydrogenase (NADP+ dependent) 2, methenyltetrahydrofolate cyclohydrolase |
| MTPAP | mitochondrial poly(A) polymerase |
| NAPG | N-ethylmaleimide-sensitive factor attachment protein, gamma |
| NDUFA9 | NADH dehydrogenase (ubiquinone) 1 alpha subcomplex, 9, 39kDa |
| NDUFB8 | NADH dehydrogenase (ubiquinone) 1 beta subcomplex, 8, 19kDa |
| NDUFC1 | NADH dehydrogenase (ubiquinone) 1, subcomplex unknown, 1, 6kDa |
| NDUFS7 | NADH dehydrogenase (ubiquinone) Fe-S protein 7, 20kDa (NADH-coenzyme Q reductase) |
| NDUFV3 | NADH dehydrogenase (ubiquinone) flavoprotein 3, 10kDa |
| NIPSNAP1 | nipsnap homolog 1 (C. elegans) |
| NIT2 | nitrilase family, member 2 |
| NOP14 | NOP14 nucleolar protein homolog (yeast) |
| NR1D1 /// THRA | nuclear receptor subfamily 1, group D, member 1 /// thyroid hormone receptor, alpha (erythroblastic leukemia viral (v-erb-a) oncogene homolog, avian) |
| NUDT19 | nudix (nucleoside diphosphate linked moiety X)-type motif 19 |
| NUDT9 | nudix (nucleoside diphosphate linked moiety X)-type motif 9 |
| PITRM1 | pitrilysin metallopeptidase 1 |
| POLG2 | polymerase (DNA directed), gamma 2, accessory subunit |
| PPOX | protoporphyrinogen oxidase |
| PRDX1 | peroxiredoxin 1 |
| QTRTD1 | queuine tRNA-ribosyltransferase domain containing 1 |
| RARS | arginyl-tRNA synthetase |
| REXO2 | REX2, RNA exonuclease 2 homolog (S. cerevisiae) |
| SFXN4 | Sideroflexin 4 |
| SLC22A4 | solute carrier family 22 (organic cation/ergothioneine transporter), member 4 |
| SLC25A15 | solute carrier family 25 (mitochondrial carrier; ornithine transporter), member 15 |
| SLC25A19 | solute carrier family 25 (mitochondrial thiamine pyrophosphate carrier), member 19 |
| SMCR7L | Smith-Magenis syndrome chromosome region, candidate 7-like |
| SUPV3L1 | suppressor of var1, 3-like 1 (S. cerevisiae) |
| TFB1M | transcription factor B1, mitochondrial |
| TFB2M | transcription factor B2, mitochondrial |
| TIMM44 | translocase of inner mitochondrial membrane 44 homolog (yeast) |
| TK2 | thymidine kinase 2, mitochondrial |
| TMEM160 | transmembrane protein 160 |
| TMLHE | trimethyllysine hydroxylase, epsilon |
| TOMM70A | translocase of outer mitochondrial membrane 70 homolog A (S. cerevisiae) |
| TXNDC12 | thioredoxin domain containing 12 (endoplasmic reticulum) |
| UQCRB | ubiquinol-cytochrome c reductase binding protein |
| UQCRQ | ubiquinol-cytochrome c reductase, complex III subunit VII, 9.5kDa |
| VARS | valyl-tRNA synthetase |
| YME1L1 | YME1-like 1 (S. cerevisiae) |
| YWHAE | tyrosine 3-monooxygenase/tryptophan 5-monooxygenase activation protein, epsilon polypeptide |

**Figure legends**

**Figure S1.** Correlation plot of real-time PCR data and microarray results. FCs were logtranformed.
