# Supplementary figures and images for: dsRNA-induced changes in gene expression profiles of primary nasal and bronchial epithelial cells from patients with asthma, rhinitis and controls
Source: Respir Res. 2014 Jan 29;15(1):9. doi: 10.1186/1465-9921-15-9 (PMC3916078; doi:10.1186/1465-9921-15-9)

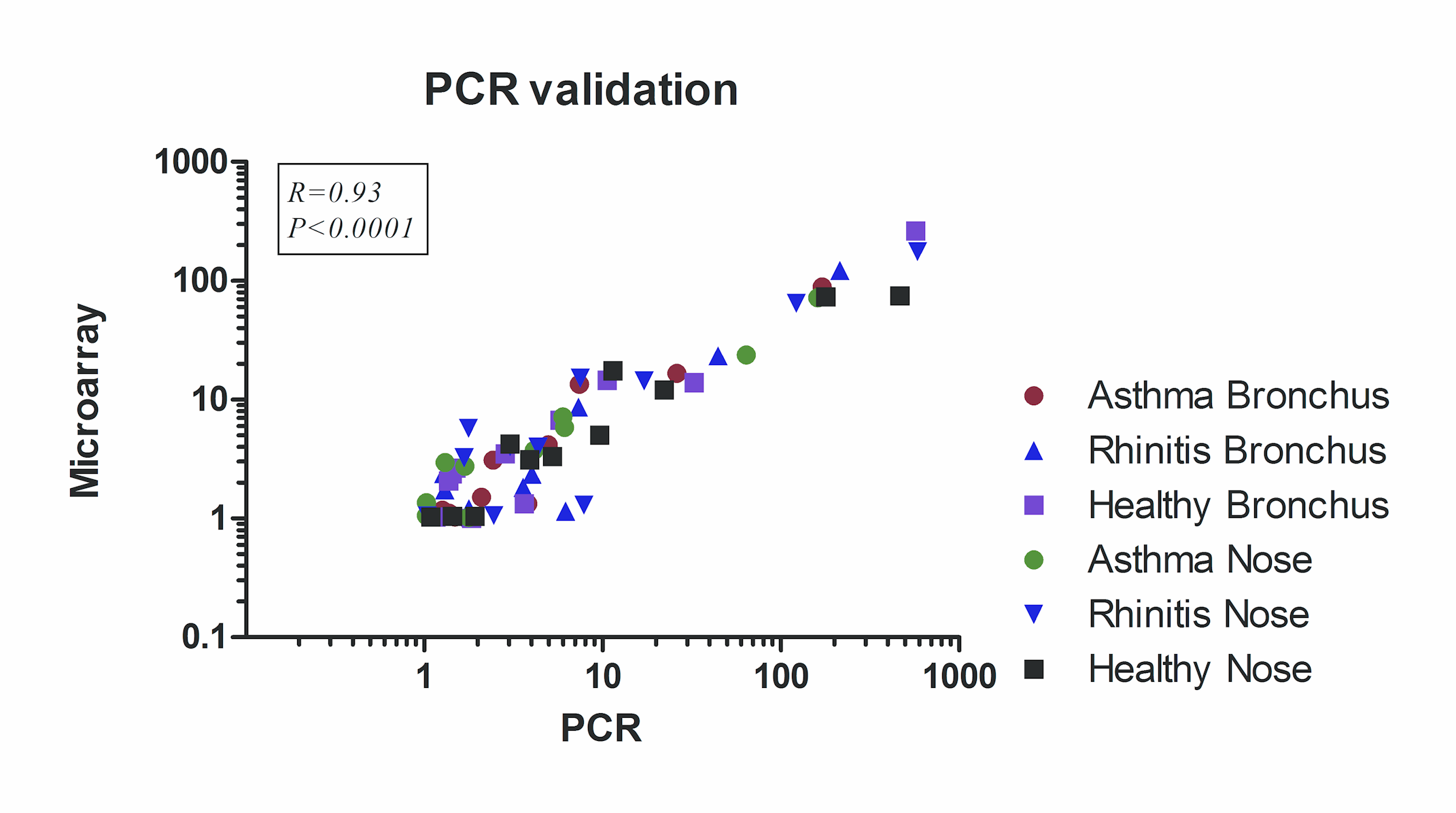

Supplement: Additional file 2: Figure S1 — Correlation plot of real-time PCR data and microarray results. FCs were logtranformed. [file 1465-9921-15-9-S2.tiff]
